# Estimation of Cross-Species Introgression Rates Using Genomic Data Despite Model Unidentifiability

**DOI:** 10.1093/molbev/msac083

**Published:** 2022-04-13

**Authors:** Ziheng Yang, Tomáš Flouri

**Affiliations:** Department of Genetics, Evolution and Environment, University College London, Gower Street, London WC1E 6BT, UK

**Keywords:** multispecies coalescent, introgression, unidentifiability, bpp, MSci, label-switching

## Abstract

Full-likelihood implementations of the multispecies coalescent with introgression (MSci) model treat genealogical fluctuations across the genome as a major source of information to infer the history of species divergence and gene flow using multilocus sequence data. However, MSci models are known to have unidentifiability issues, whereby different models or parameters make the same predictions about the data and cannot be distinguished by the data. Previous studies of unidentifiability have focused on heuristic methods based on gene trees and do not make an efficient use of the information in the data. Here we study the unidentifiability of MSci models under the full-likelihood methods. We characterize the unidentifiability of the bidirectional introgression (BDI) model, which assumes that gene flow occurs in both directions. We derive simple rules for arbitrary BDI models, which create unidentifiability of the label-switching type. In general, an MSci model with *k* BDI events has $2^{k}$ unidentifiable modes or towers in the posterior, with each BDI event between sister species creating within-model parameter unidentifiability and each BDI event between nonsister species creating between-model unidentifiability. We develop novel algorithms for processing Markov chain Monte Carlo samples to remove label-switching problems and implement them in the bpp program. We analyze real and synthetic data to illustrate the utility of the BDI models and the new algorithms. We discuss the unidentifiability of heuristic methods and provide guidelines for the use of MSci models to infer gene flow using genomic data.

## Introduction

Genomic sequences sampled from modern species contain rich historical information concerning species divergences and cross-species gene flow. In the past two decades, analysis of genomic sequence data has demonstrated the widespread nature of cross-species hybridization or introgression (Baack and Rieseberg, 2007; Harrison and Larson, 2014; Mallet et al., 2016). A number of statistical methods have been developed to infer introgression using genomic data, most of which use data summaries such as the estimated gene trees or genome-wide site-pattern counts (Degnan, 2018; Elworth et al., 2019; Jiao et al., 2021). Full-likelihood methods applied directly to multilocus sequence alignments (Wen and Nakhleh, 2018; Zhang et al., 2018; Flouri et al., 2020) allow estimation of evolutionary parameters including the timing and strength of introgression, as well as species divergence times and population sizes for modern and extinct ancestral species. These have moved the field beyond simply testing for the presence of cross-species gene flow.

Models of cross-species introgression are known to cause unidentifiability issues, whereby different introgression models make the same probabilistic predictions about the data, and cannot be distinguished by the data (Yu et al., 2012; Pardi and Scornavacca, 2015; Zhu and Degnan, 2017; Solis-Lemus et al., 2020). If the probability distributions of the data are identical under model *m* with parameters Θ and under model $m^{\prime }$ with parameters $\Theta^{\prime }$, with

(1) $$f(X\;|\;m,\Theta )=f(X\;|\;m^{\prime },\Theta^{\prime })$$

for essentially all possible data *X*, the models are unidentifiable by data *X*. Here we use the term *within-model unidentifiability* if $m=m^{\prime }$ and $\Theta \neq \Theta^{\prime }$, or *cross-model unidentifiability* if $m\neq m^{\prime }$. In the former case, two sets of parameter values in the same model are unidentifiable, whereas in the latter, two distinct models are unidentifiable. In Bayesian inference, the prior f(m,Θ) may be used to favor a particular model or set of parameters. If the prior is only vaguely informative and the posterior is dominated by the likelihood, there will be multiple modes in the posterior that are not perfectly symmetrical.

Several studies examined the unidentifiability of introgression models when gene tree topologies (either rooted or unrooted) are used as data (Pardi and Scornavacca, 2015; Zhu and Degnan, 2017; Solis-Lemus et al., 2020), and the results apply to heuristic methods based on (reconstructed) gene trees. The issue has not been studied when full-likelihood methods are applied, which operate on multilocus sequence alignments directly. Note that unidentifiability depends on the data and the method of analysis. An introgression model that is unidentifiable by gene tree topologies alone may be identifiable if gene trees with coalescent times are used (Zhu and Degnan, 2017). Similarly, a model unidentifiable using heuristic methods may be identifiable when full-likelihood methods are applied to the same data. It is thus important to study the problem when full-likelihood methods are applied, because unidentifiability by a heuristic method may reflect its inefficient use of information in the data, while unidentifiability by full-likelihood methods reflects the intrinsic difficulty of the inference problem (Zhu and Yang, 2021).

Here we focus on models of episodic introgression that assume that gene flow occurs between species at fixed time points (Wen and Nakhleh, 2018; Zhang et al., 2018; Flouri et al., 2020). These are known as multispecies-coalescent-with-introgression model (MSci; Flouri et al., 2020), hybrid species phylogenies (Kubatko, 2009), network multispecies coalescent model (NMSC; Zhu and Degnan, 2017) or multispecies network coalescent model (MSNC; Yu et al., 2012; Wen and Nakhleh, 2018; Zhang et al., 2018). Another class of models of cross-species gene flow is the continuous migration model, which assumes that migration occurs at a certain rate per generation over extended time period. This is known as the multispecies coalescent with migration (MSC+M; Jiao et al., 2021) or isolation-with-migration (IM; Hey and Nielsen, 2004; Zhu and Yang, 2012; Dalquen et al., 2017; Hey et al., 2018) model. The IM model is suitable if gene flow occurs over extended time periods, while the MSci model is preferable if gene flow occurs in short bursts of time. The IM model is in particular suitable for analyzing data from different populations of the same species. It has very different properties concerning identifiability and is not dealt with in this study.

**Fig. 1.**
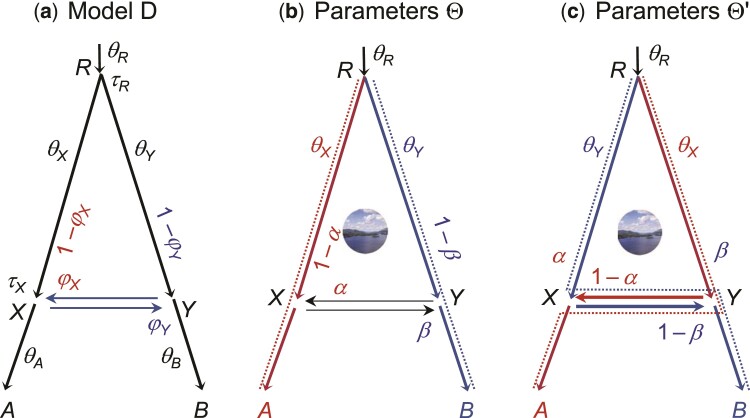
(*a*) Species tree or MSci model for two species (*A* and *B*) with a bidirectional introgression at time $\tau_{X}=\tau_{Y}$, identifying nine parameters in the model. We refer to a branch by its daughter node, so that branch *XA* is also branch *A* and is assigned the population size parameter $\theta_{A}$. Both species divergence/introgression times (τs) and population sizes (θs) are measured in the expected number of mutations per site. (*b*,*c*) Two sets of unidentifiable parameters Θ and $\Theta^{\prime }$, with $\varphi_{X}^{\prime }=1-\varphi_{X}$, $\varphi_{Y}^{\prime }=1-\varphi_{Y}$, $\theta_{X}^{\prime }=\theta_{Y}$, and $\theta_{Y}^{\prime }=\theta_{X}$, while the other five parameters ($\tau_{R},\tau_{X}=\tau_{Y},\theta_{A},\theta_{B}$, and $\theta_{R}$) are identical between Θ and $\Theta^{\prime }$. Here α and β are two numerical values for the introgression probabilities (so that $\varphi_{X}=\alpha$ in Θ while $\varphi_{X}=1-\alpha$ in $\Theta^{\prime }$). The dotted lines indicate the main routes taken by sequences sampled from species *A* and *B*, if both α and β are $\ll \frac{1}{2}$.

The bulk of the paper concerns the bidirectional introgression (BDI) model (fig. 1), which was noted to have an unidentifiability issue (Flouri et al., 2020). The BDI model posits that two species coming into contact at a certain time in the past exchange genes, while the other MSci models assume introgression only in one direction. Whether gene flow tends to occur in one direction or in both directions is an interesting empirical question that may be answered by real data analyses. Here we note that recent analyses of genomic data from North-American horned lizards (Finger et al., 2022), the erato-sara group of *Heliconius* butterflies (Thawornwattana et al., 2022), and North-American chipmunks (Ji et al., 2021) have identified BDI events, both between sister species and between nonsister species (see also an example later). In the *Anopheles gambiae* group of African mosquitoes, introgression between *A. gambiae* and *A. arabiensis* in both directions was suspected, but detailed analyses detected gene flow from *A. arabiensis* to *A. gambiae* only but not in the opposite direction (Thawornwattana et al., 2018). In another example, Banker et al. (2022) detected bidirectional introgression (with different rates) between *Mus spretus* and wild populations of *M. m. domesticus* from Europe, despite considerable postzygotic reproductive isolation between the species. At any rate, BDI is one of the most plausible introgression models and appears to be one of the most common forms of cross-species gene flow. The unidentifiability of MSci models with unidirectional introgression (UDI) is simpler, and we defer its discussion to the Discussion section. Similarly, we discuss unidentifiability of heuristic methods later.

The basic BDI model between two species (fig. 1) involves nine parameters, with $\Theta =(\theta_{A},\theta_{B},\theta_{X},\theta_{Y},\theta_{R},\tau_{R},\tau_{X},\varphi_{X},\varphi_{Y})$. An introgression model is similar to a species tree except that it includes horizontal branches representing lateral gene flow across species. Besides speciation nodes representing species divergences, there are hybridization nodes representing introgression events as well. While a speciation node has one parent and two daughters, a hybridization node has two parents and one daughter. The “introgression probabilities” or “admixture proportions” (φ and 1−φ) specify the contributions of the two parental populations to the hybrid species. When we trace the genealogical history of a sample of sequences from the modern species backwards in time and reach a hybridization node, each of the sequences takes the two parental paths with probabilities φ and 1−φ. There are thus three types of parameters in an MSci model: the times of species divergence and introgression (τs), the (effective) population sizes of modern and ancestral species (θs), and the introgression probabilities (φs). Both the divergence times (τs) and population sizes (θs) are measured in the expected number of mutations per site.

The BDI model, in the case of two species (fig. 1), is noted to have an unidentifiability issue (Flouri et al., 2020). Let $\Theta^{\prime }$ be a set of parameters with the same values as Θ except that $\varphi_{X}^{\prime }=1-\varphi_{X}$, $\varphi_{Y}^{\prime }=1-\varphi_{Y}$, $\theta_{X}^{\prime }=\theta_{Y}$, and $\theta_{Y}^{\prime }=\theta_{X}$. Then $f(G\;|\;\Theta )=f(G\;|\;\Theta^{\prime })$ for any gene tree *G* (fig. 1*b* and *c*). Here *G* represents both the gene tree topology and branch lengths (coalescent times). We assume multiple sequences sampled per species at the same locus (see Discussion for unidentifiability caused by sampling only one sequence per species). Thus for every point Θ in the parameter space, there is a “mirror” point $\Theta^{\prime }$ with exactly the same likelihood. With Θ, the *A* sequences take the left (upper) path at *X* and enter population *RX* with probability $1-\varphi_{X}$, coalescing at the rate $2/\theta_{X}$, while with $\Theta^{\prime }$, the same *A* sequences may take the right (horizontal) path and enter population *RY* with probability $\varphi_{X}^{\prime }=1-\varphi_{X}$, coalescing at the rate $2/\theta_{Y}^{\prime }=2/\theta_{X}$. The differences between Θ and $\Theta^{\prime }$ are in the labeling, with “left” and *X* under Θ corresponding to “right” and *Y* under $\Theta^{\prime }$, but the probabilities involved are the same. The same argument applies to sequences from *B* going through node *Y*, and to any numbers of sequences from *A* and *B* considered jointly. Thus $f(G\;|\;\Theta )=f(G\;|\;\Theta^{\prime })$ for essentially all *G*. If the priors on $\varphi_{X}$ and $\varphi_{Y}$ are symmetrical, say φ∼beta(α,α), the posterior density will satisfy $f(\Theta \;|\;X)=f(\Theta^{\prime }\;|\;X)$ for all *X*. Otherwise the “twin towers” in the posterior may not have exactly the same height.

The situation is very similar to the label-switching problem in Bayesian clustering (Richardson and Green, 1997; Celeux et al., 1998; Stephens, 2000; Jasra et al., 2005). Consider data $X=\{x_{i}\}$ as a sample from a mixture of two normal distributions, $N\;(\mu_{1},1)$ and $N\;(\mu_{2},1)$, with the mixing proportions $p_{1}$ and $p_{2}=1-p_{1}$. Let $\Theta =(p_{1},\mu_{1},\mu_{2})$ be the parameter vector. Then $\Theta^{\prime }=(p_{2},\mu_{2},\mu_{1})$ will have exactly the same likelihood, with $f(X\;|\;\Theta )=f(X\;|\;\Theta^{\prime })$ for essentially all data *X*, and Θ and $\Theta^{\prime }$ are unidentifiable. Suppose the data suggest two groups in proportions 10% and 90%, with means 100 and 1, so that there are two peaks in the posterior, around $\Theta \;:\;p_{1}=0.1$, $\mu_{1}=100$, $\mu_{2}=1$ and $\Theta^{\prime }\;:\;p_{1}^{\prime }=0.9$, $\mu_{1}^{\prime }=1$, $\mu_{2}^{\prime }=100$. In a Bayesian cluster analysis using Markov chain Monte Carlo (MCMC), the Markov chain may visit both peaks, effectively switching the labels “group 1” and “group 2” and changing the definitions of parameters in the same MCMC run. This is known as a *label-switching problem*. One may process the MCMC sample, and reflect each $\Theta^{\prime }$ with $p_{1}^{\prime }>\frac{1}{2}$ to its mirror point Θ, to fix the label-switching (but see later for problems involved with imposing such simple constraints). In other words, we may apply a *relabeling algorithm* to postprocess the MCMC sample to fix the label-switching issue.

**Fig. 2.**
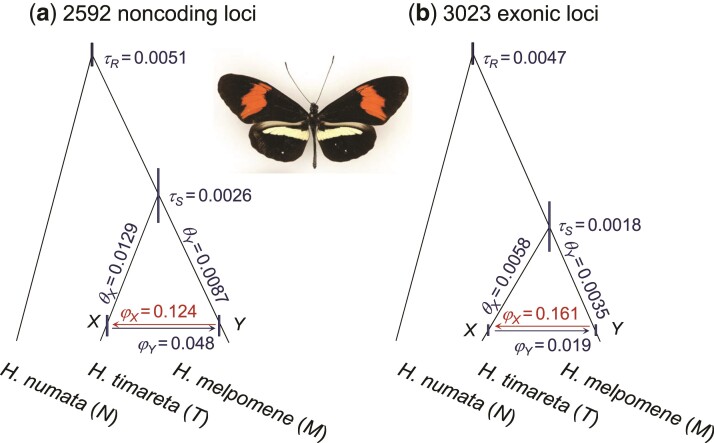
Species tree or BDI model for *Heliconius melpomene*, *H. timareta*, and *H. numata*. The branches are drawn to represent the posterior means of divergence/introgression times obtained from bpp analysis of (*a*) the 2,592 noncoding and (*b*) the 3,023 exonic loci from chromosome 1, while the node bars represent the 95% HPD CIs. See table 1 for estimates of all parameters. Photo of *H. timareta* courtesy of James Mallet.

**Fig. 3.**
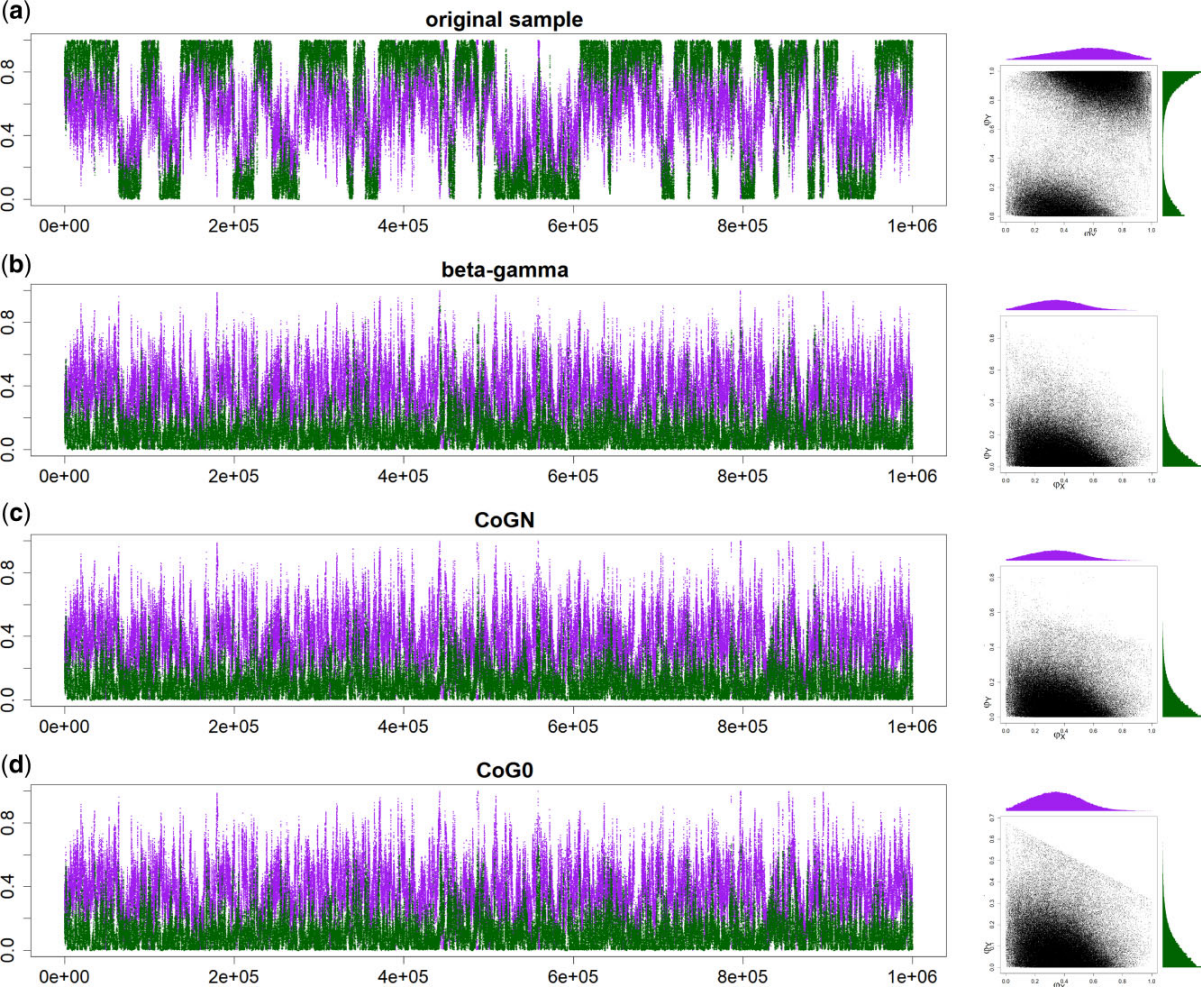
Trace plots of MCMC samples and 2-D scatter plots for parameters $\varphi_{X}$ (purple) and $\varphi_{Y}$ (green) (*a*) before and (*b*–*d*) after the postprocessing of the MCMC sample in the bpp analysis of the first 500 noncoding loci from chromosome 1 of the *Heliconius* data under the MSci model of figure 2. The three algorithms used are (*b*) β–γ, (*c*) CoG${}_{N}$, and (*d*) CoG${}_{0}$.

As an example of the label-switching issues in the BDI model, consider the MCMC analysis using bpp of the first 500 noncoding loci on chromosome 1 from three *Heliconius* butterfly species: *H. melpomene*, *H. timareta*, and *H. numata* (Edelman et al., 2019; Thawornwattana et al., 2022) (fig. 2*a*). Figure 3*a* shows the trace plots for parameters $\varphi_{X}$ and $\varphi_{Y}$ from an MCMC run. The Markov chain moves between two peaks, centered around $\left(\varphi_{X},\varphi_{Y})=(0.35,0.1\right)$ and (0.65,0.9), respectively. In effect, the algorithm is switching between Θ and $\Theta^{\prime }$ and changing the definition of parameters during the same MCMC run. This is a label-switching problem, as occurs in Bayesian clustering. The usual practice of estimating parameters by their posterior means calculated using the MCMC sample (0.54 for $\varphi_{X}$ and 0.62 for $\varphi_{Y}$ in fig. 3*a*) and constructing the credibility intervals is inappropriate. Indeed the posterior distribution of Θ is exactly symmetrical with twin towers, and if the chain is run long enough, the sample means of $\varphi_{X}$ and $\varphi_{Y}$ will be exactly $\frac{1}{2}$, irrespectively of what values may fit the data well. The results are similar when the first 500 exonic loci are analyzed, in which the Markov chain moves between two towers centered around (0.3,0.1) and (0.7,0.9) (supplementary fig. S1*a*, Supplementary Material online).

**Table 1.** Posterior means and 95% HPD CIs (in parentheses) for parameters in the BDI model of figure 2 for the *Heliconius* data.

	First 500 loci	Chromosome 1	All autosomal loci
Noncoding	L=500	L=2,592	L=31,166
$\tau_{R}$	4.73 (4.33, 5.13)	5.10 (4.89, 5.30)	5.03 (4.97, 5.10)
$\tau_{S}$	3.12 (2.05, 4.19)	2.58 (2.12, 3.05)	2.50 (2.35, 2.65)
$\tau_{X}=\tau_{Y}$	0.62 (0.21, 1.02)	0.25 (0.09, 0.40)	0.08 (0.05, 0.11)
$\theta_{M}$	1.50 (0.62, 2.34)	0.69 (0.35, 1.10)	0.22 (0.14, 0.32)
$\theta_{T}$	2.55 (1.40, 3.74)	1.23 (0.65, 1.84)	0.22 (0.14, 0.31)
$\theta_{N}$	15.1 (12.0, 18.5)	23.0 (20.3, 25.7)	9.58 (9.36, 9.80)
$\theta_{R}$	5.08 (4.12, 6.05)	5.74 (5.23, 6.24)	6.57 (6.40, 6.74)
$\theta_{S}$	4.62 (1.85, 7.40)	6.92 (5.48, 8.37)	7.75 (7.23, 8.26)
$\theta_{X}$	11.40 (2.83, 21.2)	12.90 (7.35, 19.6)	11.7 (10.4, 13.1)
$\theta_{Y}$	6.78 (2.42, 11.6)	8.74 (5.69, 12.0)	8.52 (7.50, 9.53)
$\varphi_{X}$	0.354 (0.022, 0.664)	0.124 (0.007, 0.243)	0.036 (0.001, 0.064)
$\varphi_{Y}$	0.104 (0.000, 0.306)	0.048 (0.000, 0.139)	0.074 (0.032, 0.117)
Exonic	L=500	L=3,023	L=36,138
$\tau_{R}$	4.39 (3.98, 4.81)	4.71 (4.54, 4.88)	5.04 (4.98, 5.10)
$\tau_{S}$	1.95 (1.07, 2.82)	1.78 (1.38, 2.19)	1.54 (1.43, 1.64)
$\tau_{X}=\tau_{Y}$	0.20 (0.03, 0.37)	0.13 (0.05, 0.24)	0.05 (0.04, 0.07)
$\theta_{M}$	0.38 (0.08, 0.70)	0.32 (0.14, 0.52)	0.14 (0.11, 0.16)
$\theta_{T}$	0.79 (0.13, 1.28)	0.63 (0.32, 0.94)	0.13 (0.10, 0.15)
$\theta_{N}$	11.2 (9.11, 13.5)	12.4 (11.4, 13.4)	7.80 (7.65, 7.95)
$\theta_{R}$	5.76 (4.83, 6.70)	6.68 (6.24, 7.11)	7.72 (7.57, 7.87)
$\theta_{S}$	5.31 (3.38, 7.36)	7.50 (6.51, 8.49)	9.99 (9.64, 10.4)
$\theta_{X}$	8.04 (1.67, 15.4)	5.80 (3.60, 8.36)	6.63 (6.12, 7.17)
$\theta_{Y}$	4.03 (0.60, 7.51)	3.49 (2.56, 4.50)	5.20 (4.81, 5.59)
$\varphi_{X}$	0.280 (0.002, 0.547)	0.161 (0.070, 0.264)	0.045 (0.022, 0.069)
$\varphi_{Y}$	0.116 (0.000, 0.318)	0.019 (0.000, 0.056)	0.016 (0.000, 0.037)

note.—Estimates of τs and θs are multiplied by $10^{3}$. MCMC samples are processed using the β–γ algorithm before they are summarized.

Results such as those of fig. 3*a* and supplementary fig. S1*a*, Supplementary Material online raise two questions. First, what are the rules concerning the unidentifiability of general BDI models with, for example, more than two species on the species tree and more than one BDI event, or if the BDI event involves nonsister species. Second, how do we deal with the problem of label-switching and make the models useful for real data analyses? We address those two problems in this paper. We study the unidentifiability issue of BDI models for an arbitrary number of species with an arbitrary species tree, when a full-likelihood method is applied to multilocus sequence data. It has been conjectured that an MSci model is identifiable by full-likelihood methods using data of multilocus sequence alignments if and only if it is identifiable when the data consist of gene trees with coalescent times (Flouri et al., 2020). We make use of this conjecture and consider two BDI models to be unidentifiable if and only if they generate the same distribution of gene trees with coalescent times. We emphasize that the unidentifiability discussed here affects all methods of inference using genomic sequence data, including heuristic methods based on summary statistics (see Discussion). We identify general rules for the unidentifiability of the BDI models. We then develop new relabeling algorithms for postprocessing the MCMC samples generated from a Bayesian analysis under the BDI model to remove the label-switching. The algorithms remove the label-switching issues but do not remove the unidentifiability, which is the nature of the model and data. While in the clustering problem, the labels “group 1” and “group 2” are of no significance, Θ and $\Theta^{\prime }$ under the unidentifiable BDI models may represent different biological hypotheses, and one may want to choose between them. This is discussed later in the subsection “Estimation of introgression probabilities despite unidentifiability” in Discussion. Our efforts make the BDI models usable for real data analysis despite their unidentifiability. We use the bpp program (Flouri et al., 2018) to analyze synthetic datasets as well as genomic data from *Heliconius* butterflies to demonstrate the utility of the BDI models and the new algorithms. After we have dealt with the BDI models, we discuss the unidentifiability of UDI models and of heuristic methods.

## Theory

### The Rule of Unidentifiability of BDI Models

In full-likelihood implementations of the MSci model, the gene tree *G* for any given sample of sequences from the modern species represents the complete history of coalescence and introgression events for the sample, including the gene tree topology, the coalescent times, as well as the parental path taken by each sequence at each hybridization node (e.g., Jiao et al., 2021, eq. 14). The probability distribution of the gene tree *G* depends on the species tree, species divergence times (τs), the population sizes (θs) which determine the coalescent rates in the different populations (2/θ), and the introgression probabilities at the hybridization nodes (φ). It does not depend on the labels attached to the internal nodes in the species tree.

**Fig. 4.**
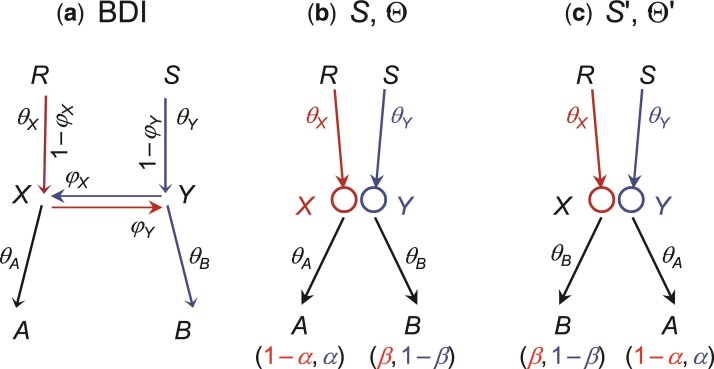
A part of a species tree (MSci model) for illustrating the rule of BDI unidentifiability. (*a*) In the BDI model, species *RXA* and *SYB* exchange migrants at time $\tau_{X}=\tau_{Y}$. Treat *X* and *Y* as one node with left parent *RX* with population size $\theta_{X}$ and right parent *SY* with population size $\theta_{Y}$. When a sequence from *A* reaches *XY*, it takes the left and right parental paths with probabilities $1-\varphi_{X}$ and $\varphi_{X}$, respectively. When a sequence from *B* reaches *XY*, it goes left and right with probabilities $\varphi_{Y}$ and $1-\varphi_{Y}$, respectively. (*b*,*c*) Placing the two daughters in the order (A,B) as in Θ or (B,A) as in $\Theta^{\prime }$ does not affect the distribution of gene trees, and constitutes unidentifiable towers in the posterior space. If *X* and *Y* are sister species and have the same mother node (with *R* and *S* to be the same node), the unidentifiability is within-model; otherwise it is cross-model.

Consider a part of the species tree or MSci model where species *A* and *B* exchange migrants at time $\tau_{X}=\tau_{Y}$ (fig. 4). To study the backwards-in-time process of coalescent and introgression, which gives the probability density of the gene tree f(G|S,Θ), we can treat nodes *X* and *Y* as one node, *XY*. When sequences from *A* reach node *XY*, each of them has probability $1-\varphi_{X}$ of taking the left parental path (*RX*) and probability $\varphi_{X}$ of taking the right parental path (*SY*). Similarly when sequences from *B* reach node *XY*, they have probabilities $\varphi_{Y}$ and $1-\varphi_{Y}$ of taking the left (*RX*) and right (*SY*) parental paths, respectively. If we swap branches *A* and *B*, carrying with them their population size parameters (θ) and introgression probabilities (φ), the probability density of the gene trees remains unchanged. Thus the species tree-parameter combinations (S,Θ) and ($S^{\prime },\Theta^{\prime }$) of figure 4*b* and *c* give exactly the same probability distribution,

(2) $$f(G\;|\;S,\Theta )=f(G\;|\;S^{\prime },\Theta^{\prime }),\;\text{for every gene tree}\;G.$$

In other words, (S,Θ) and ($S^{\prime },\Theta^{\prime }$) are unidentifiable (see eq. 1).

Note that the processes of coalescent and introgression before reaching nodes *A* and *B* (with time running backwards) are identical between Θ and $\Theta^{\prime }$, as are the processes past nodes *X* and *Y*. Thus the rule applies if each of *A* and *B* is a subtree, with introgression events inside, or if there are introgression events involving a descendant of *A* and a descendant of *B*.

If *A* and *B* are sister species or the parents *R* and *S* are one node in the species tree, the species trees (A,B) and (B,A) will be the same so that $S=S^{\prime }$ in equation (2). Then Θ and $\Theta^{\prime }$ (fig. 4) will be two sets of parameter values in the same model and we have a case of within-model unidentifiability. Otherwise the unidentifiability is cross-model.

### Canonical Cases of BDI Models

Here we study major BDI models to illustrate the rule of unidentifiability and to provide reference for researchers who may apply those models to analyze genomic datasets.

If we add subtrees onto branches *XA*, *YB*, or the root branch *R* in the two-species tree of figure 1*a*, so that the BDI event remains to be between two sister species, the model will exhibit within-model parameter unidentifiability (supplementary fig. S2, Supplementary Material online), just like the basic model of figure 1*a*.

If the BDI event is between nonsister species, the model exhibits cross-model unidentifiability. Supplementary figure S3*a* and *a*', Supplementary Material online show a model with a BDI event between cousins, while in supplementary figure S3*b* and *b*', Supplementary Material online, the two species involved in the BDI event are more distantly related.

Supplementary figures S4 *a*, S4*b* and S4*c*, Supplementary Material online show three models each with a BDI event between nonsister species. In supplementary figure S4*a*, Supplementary Material online, *X* and *Y* are nonsister species on the original binary species tree. In supplementary figure S4*b* and *c*, Supplementary Material online, *X* and *Y* are nonsister species because there are introgression events involving branches *RX* and/or *RY*. In all three cases, there is cross-model unidentifiability, with the twin towers shown in supplementary figure S4*a*', *b*' and *c*', Supplementary Material online.

The case of two nonsister BDI events for three species is illustrated in supplementary figure S5, Supplementary Material online. According to our rule, there are four unidentifiable models in the posterior, with parameter mappings shown in supplementary figure S5, Supplementary Material online. One way of seeing that the four models are equivalent or unidentifiable is to assume that the introgression probabilities ($\varphi_{X}$, $\varphi_{Y}$, $\varphi_{Z}$, and $\varphi_{W}$) are all $<\frac{1}{2}$, and then work out the major routes taken when we trace the genealogical history of sequences sampled from modern species. In such cases, all four models of supplementary figure S5, Supplementary Material online predict the following: most sequences from *A* will take the route *RZ* at node *ZW* with probability 1−γ; most sequences from *B* will take the route *WX* at node *XY* (with probability 1−α), then the route *WS* at node *ZW* (with probability 1−δ), before reaching *RS*; and most sequences from *C* will take the route *SY* at node *XY* (with probability 1−β), before reaching *RS*. Of course the four models are unidentifiable whatever values the introgression probabilities take. Those models have been used to analyze genomic data from Texas Horned Lizards (*Phrynosoma cornutum*) (Finger et al., 2022, figure S9).

**Fig. 5.**
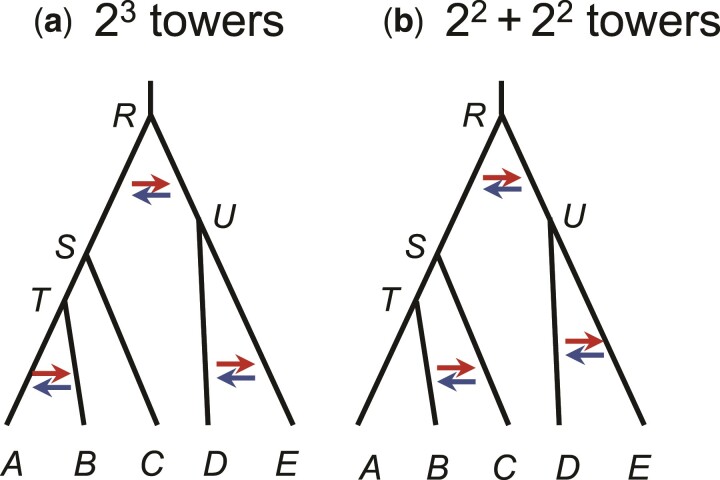
Two species trees (MSci models) for five species each with three BDI events. (*a*) Three BDI events between sister species create $2^{3}=8$ within-model towers in the posterior. (*b*) Two BDI events between sister species and one BDI event between nonsister species create two unidentifiable models each with four within-model unidentifiable towers in the posterior space.

Figure 5 shows two models for five species, each model involving three BDI events. In figure 5*a*, all three BDI events involve sister species, so that there are $2^{3}=8$ unidentifiable within-model towers in the posterior. In figure 5*b*, one BDI event involves nonsister species while two involve sister species, so that there are two unidentifiable models, each of which has four unidentifiable within-model towers in the posterior.

In general, if there are *m* BDI events between sister species and *n* BDI events between nonsister species, there will be $2^{n}$ unidentifiable models, each having $2^{m}$ within-model unidentifiable towers.

### Unidentifiability of Double-BDI Models

**Fig. 6.**
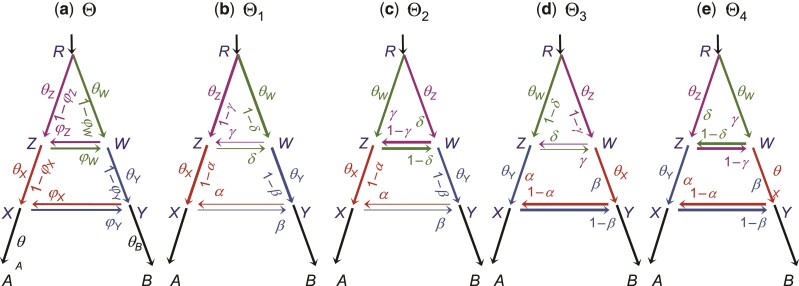
Species trees (MSci models) for two species (*A* and *B*) with double-BDI events creating four within-model towers, represented by $\Theta_{1}$, $\Theta_{2}$, $\Theta_{3}$, and $\Theta_{4}$. (*a*) The model involves 14 parameters: 7 θs, 3 τs, and 4  φs, with eight of them involved in the label-switching unidentifiability, $\Theta =(\varphi_{X},\varphi_{Y},\theta_{X},\theta_{Y},\varphi_{Z},\varphi_{W},\theta_{Z},\theta_{W})$. (*b*–*e*) Four unidentifiable towers showing the mappings of parameters (eq. 3). To apply the rule of figure 4, we treat each pair of BDI nodes as one node, so that *X* and *Y* have the same node *ZW* as the parent, and the unidentifiability caused by the BDI event at node *XY* is within-model, as is the unidentifiability for the BDI event at node *ZW*.

Figure 6 *a* shows two BDI events between species *A* and *B*, which occurred at times $\tau_{X}=\tau_{Y}$ and $\tau_{Z}=\tau_{W}$, respectively. To apply the rule of figure 4, we treat *Z* and *W* as one node so that *X* and *Y* are considered sister species. There are then four within-model unidentifiable towers in the posterior space, shown as $\Theta_{1}$−$\Theta_{4}$ in figure 6. The parameter mappings are given in the table above.

**Table:** 

Θ	*φ*_*X*_	*φ*_*Y*_	*θ*_*X*_	*θ*_*Y*_	*φ*_*Z*_	*φ*_*W*_	*θ*_*Z*_	*θ*_*W*_
Θ_1_ : *φ*_*X*_ < $\frac{1}{2}$, *φ*_*Z*_ < $\frac{1}{2}$	*α*	*β*	*θ*_*X*_	*θ*_*Y*_	*γ*	*δ*	*θ*_*Z*_	*θ*_*W*_
Θ_2_ : *φ*_*X*_ < $\frac{1}{2}$, *φ*_*Z*_ > $\frac{1}{2}$	*α*	*β*	*θ*_*X*_	*θ*_*Y*_	1 − *γ*	1 − *δ*	*θ*_*W*_	*θ*_*Z*_
Θ_3_ : *φ*_*X*_ > $\frac{1}{2}$, *φ*_*W*_ < $\frac{1}{2}$	1 − *α*	1 − *β*	*θ*_*Y*_	*θ*_*X*_	*δ*	*γ*	*θ*_*W*_	*θ*_*Z*_
Θ_4_ : *φ*_*X*_ > $\frac{1}{2}$, *φ*_*W*_ > $\frac{1}{2}$	1 − *α*	1 − *β*	*θ*_*Y*_	*θ*_*X*_	1 − *δ*	1 − *γ*	*θ*_*Z*_	*θ*_*W*_

(3)

In general, with *k* BDI events between two species, which occurred at different time points in the past, there will be $2^{k}$ unidentifiable within-model towers in the posterior. There may be little information in practical datasets to estimate so many parameters: if all sequences have coalesced before they reach the ancient introgression events near the root of the species tree, the introgression probabilities (φs) and the associated population sizes (θs) will be nearly impossible to estimate. Thus we do not consider more than two BDI events between two species. Note that even the model with one BDI event is not identifiable by heuristic methods that use gene tree topologies only. A small simulation is conducted to illustrate the feasibility of applying the double-BDI model (fig. 6) to genomic datasets; see Results.

### Addressing Label-switching Issues and Difficulties with Identifiability Constraints

According to our rule, MSci models with BDI events can create both within-model and cross-model unidentifiability. Cross-model unidentifiability is relatively simple to identify and deal with. If the MCMC is run with the MSci model fixed (Flouri et al., 2020), only one of the models (e.g., model $S_{1}$ with parameters $\Theta_{1}$ in supplementary fig. S5, Supplementary Material online) is visited in the chain. One can then summarize the posterior distribution for parameters under that model (which may be smooth and single-moded), and the posterior summary may be mapped onto the other unidentifiable models according to the rule. See Finger et al. (2022) for such an application of BDI models of supplementary figure S5, Supplementary Material online. If the MCMC is trans-model and visits different models in the chain (Wen and Nakhleh, 2018; Zhang et al., 2018), the posterior space is symmetrical between the unidentifiable models (such as models $S_{1}$–$S_{4}$ of supplementary fig. S5, Supplementary Material online). However, such symmetry is unlikely to be achieved in the MCMC sample due to well-known mixing difficulties of trans-model MCMC algorithms. One may choose to focus on one of the models (e.g., $S_{1}$ of supplementary fig. S5, Supplementary Material online) and postprocess the MCMC sample to map the sample onto the chosen model before producing the within-model posterior summary. Oftentimes the MCMC may explore the within-model posterior space very well, despite difficulties of moving from one model to another. In all cases, the researcher has to be aware of the unidentifiable models which are equally good explanations of the genetic data (see Discussion).

Our focus here is on within-model unidentifiability created by BDI events between sister species. When there are multiple modes in the posterior, each mode may offer a sensible interpretation of the data, but it is inappropriate to merge MCMC samples from different modes, or to construct posterior summaries such as the posterior means and credibility intervals (CIs) using MCMC samples that traverse different modes. It is instead more appropriate to summarize the samples for each mode.

A common strategy for removing label-switching is to apply so-called *identifiability constraints*. In the simple BDI model of figure 1, any of the following constraints may be applicable: $\varphi_{X}<\frac{1}{2}$, $\varphi_{Y}<\frac{1}{2}$, and $\theta_{X}<\theta_{Y}$. Such identifiability constraints may be imposed during the MCMC or during postprocessing of the MCMC samples. As discussed previously (Celeux et al., 1998; Stephens, 2000), such a constraint may be adequate if the posterior modes are well separated, but may not work well otherwise. For example, if $\varphi_{X}$ is far away from $\frac{1}{2}$ in all MCMC samples, it will be simple to postprocess the MCMC sample to impose the constraint $\varphi_{X}<\frac{1}{2}$. This is the case in analyses of the large datasets in this paper, for example, when all noncoding and exonic loci from chromosome 1 of the *Heliconius* data are analyzed (table 1). However, when the posterior modes are not well-separated (either because the true parameter value is close to the boundary defined by the inequality or because the data lack information so that the CIs are wide), different identifiability constraints can lead to very different parameter posteriors (Richardson and Green, 1997), and an appropriate constraint may not exist. Imposing identifiability constraints may then generate posterior distributions over-represented near the boundary, with seriously biased posterior means (Celeux et al., 1998; Stephens, 2000). For example, $\varphi_{X}$ may have substantial density mass both below and above $\frac{1}{2}$, and imposing the constraint $\varphi_{X}<\frac{1}{2}$ will artificially generate high density mass close to $\varphi_{X}=\frac{1}{2}$. Similarly the posterior distributions of $\theta_{X}$ and $\theta_{Y}$ may overlap, so that the constraint $\theta_{X}<\theta_{Y}$ may not be appropriate.

### New Algorithms to Process MCMC Samples from the BDI Model to Remove Label Switching

One approach to dealing with label-switching problems in Bayesian clustering is *relabeling*. The MCMC is run without any constraint, and the MCMC sample is then postprocessed to remove label switching, by attempting to move each point in the MCMC sample to its alternative unidentifiable positions in order to, as far as possible, make the marginal posterior distributions smooth and unimodal (Celeux et al., 1998; Stephens, 2000). The processed sample is then summarized to generate the posterior of the parameters. Here we follow this strategy and implement three relabeling algorithms to postprocess the MCMC samples generated under the BDI model.

Let $\Theta =(\varphi_{X},\varphi_{Y},\theta_{X},\theta_{Y})$, which has a mirror point $\Theta^{\prime }=(\varphi_{X}^{\prime },\varphi_{Y}^{\prime },\theta_{X}^{\prime },\theta_{Y}^{\prime })=(1-\varphi_{X},1-\varphi_{Y},\theta_{Y},\theta_{X})$ (fig. 1). The other parameters in the model are not involved in the unidentifiability and are simply copied along. Let $\Theta_{t}$, t=1,…,N, be the *N* samples of parameters generated by the MCMC algorithm. Each sample is a point in the 4-D space. Let $z_{t}$ be a transform for point *t*, with $z_{t}(\Theta_{t})=\Theta_{t}$ to be the original point, and $z_{t}(\Theta_{t})=\Theta_{t}^{\prime }$ to be the transformed or mirror point (fig. 1*b* and *c*). With a slight abuse of notation, we also treat $z_{t}$ as an indicator, with $z_{t}=0$ and 1 representing $\Theta_{t}$ and $\Theta_{t}^{\prime }$, respectively. For each sample *t*, we choose either the original point or its mirror point, to make the posterior of the parameters look smooth and single-moded as far as possible. The first two algorithms, called center-of-gravity algorithms CoG${}_{0}$ and CoG${}_{N}$, loop through two steps.

*Algorithms CoG${}_{0}$ and CoG${}_{N}$.* Initialize. For each point *t*, t=1,…,N, pick either the original point ($\Theta_{t}$) or its mirror point ($\Theta_{t}^{\prime }$). We set $z_{t}$ to 0 (for the original point $\Theta_{t}$) if $\varphi_{X}+\varphi_{Y}<1$ or 1 (for the mirror point $\Theta_{t}^{\prime }$) otherwise.

- Step 1. Determine the center of gravity, given by the sample means of the parameters, $\mu =({\bar{\varphi }}_{X},{\bar{\varphi }}_{Y},{\bar{\theta }}_{X},{\bar{\theta }}_{Y})$.
- Step 2. For each point t=1,…,N, compare the current and its mirror positions and choose the one closer to the center of gravity (μ).

In step 2, we use the Euclidean distance

(4) $$d_{0}(\Theta_{t},\mu )={\left[\sum_{j}^{4}(\xi_{j}-\mu_{j}{)}^{2}\right]}^{1/2},$$

where $\xi_{j}$ are the four parameters in $\Theta_{t}$: $\varphi_{X},\varphi_{Y},\theta_{X},\theta_{Y}$. This is algorithm CoG${}_{0}$.

If we consider different scales in the different dimensions (for example, $\varphi_{X}$ and $\theta_{X}$ may have very different posterior variances), we can calculate the sample variances ν (in addition to the sample means μ) in step 1 and use them as weights to normalize the differences in step 2, with

(5) $$d_{N}(\Theta_{t},\mu )={\left[\sum_{j}^{4}\frac{1}{\nu_{j}}(\xi_{j}-\mu_{j}{)}^{2}\right]}^{1/2}.$$

We refer to this as algorithm CoG${}_{N}$.

Each MCMC sample point $\Theta_{t}$ can be in either of two positions (represented by $z_{t}=0$ or 1). Step 1 calculates the center of attraction (μ), which represents the current “location of most points.” Step 2 then moves each point to its mirror position it is closer to the current center of attraction. If there are only two modes in the posterior (due to label switching) but no other modes, one of the unidentifiable modes will become the center of attraction and all points will move to its neighborhood as the algorithm progresses. Which of the two modes becomes the center of attraction is arbitrary, influenced by the initial positions when the algorithm runs.

The third algorithm, called the β–γ algorithm, follows the relabeling algorithm for Bayesian clustering of Stephens (2000). We use maximum likelihood (ML) to fit the sample $\left\{\Theta_{t}\right\}$ to independent beta distributions for $\varphi_{X}$ and $\varphi_{Y}$ and gamma distributions for $\theta_{X}$ and $\theta_{Y}$:

(6) $$\begin{array}{rl} \;f(\Theta ;\omega ) & =b(\varphi_{X};p_{X},q_{X})\cdot b(\varphi_{Y};p_{Y},q_{Y})\\ & \;\times g(\theta_{X};a_{X},b_{X})\cdot g(\theta_{Y};a_{Y},b_{Y}), \end{array}$$

where

(7) $$\begin{array}{rl} b(\xi ;p,q) & =\frac{1}{B(p,q)}\xi^{\;p-1}(1-\xi {)}^{q-1},\\ g(\xi ;a,b) & =\frac{b^{a}}{\Gamma (a)}\xi^{a-1}e^{-b\xi } \end{array}$$

are the beta and gamma densities and where ω= ($p_{X}$, $q_{X}$, $p_{Y}$, $q_{Y}$, $a_{X},b_{X},a_{Y},b_{Y}$) is the vector of parameters in those densities.

The log likelihood, as a function of the parameters ω and the transforms $z=\{z_{t}\}$, is

(8) $$\ell (\omega ,z)=\sum_{t}^{N}\ell_{t}(\omega ,z_{t}(\Theta_{t}))=\sum_{t}^{N}\log\;f(z_{t}(\Theta_{t});\omega ),$$

where the density *f* is given in equation (6).

We have implemented the following iterative algorithm to estimate ω and *z* by maximizing ℓ.

*Algorithm β–γ.* Initialize $z_{t},t=1,\ldots ,N$. As before, we set $z_{t}$ to 0 (for $\Theta_{t}$) if $\varphi_{X}+\varphi_{Y}<1$ or 1 (for $\Theta_{t}^{\prime }$) otherwise.

- Step 1. Choose $\hat{\omega }$ to maximize the log likelihood ℓ (eq. 8) with the transforms *z* fixed.
- Step 2. For t=1,…,N, choose $z_{t}=0$ or 1 to maximize $\ell_{t}(\hat{\omega },z_{t}(\Theta_{t}))$ with $\omega =\hat{\omega }$ fixed. In other words, compare $\Theta_{t}$ and $\Theta_{t}^{\prime }$ and choose the one that better fits the beta and gamma distributions.

Step 1 fits two beta and two gamma distributions by ML and involves four separate 2-D optimization problems. The maximum-likelihood estimates (MLEs) of *p* and *q* for the beta distribution b(ξ;p,q) are functions of $\sum_{t}\log\;\xi_{t}$ and $\sum_{t}\log\;(1-\xi_{t})$, whereas the MLEs of *a* and *b* for the gamma distribution g(ξ;a,b) are functions of $\sum_{t}\xi_{t}$ and $\sum_{t}\log\;\xi_{t}$. These optimization problems are simple, which we solve using the BFGS algorithm in the paml program (Yang, 2007). Step 2 involves *N* independent optimization problems, each comparing two points ($z_{t}=0$ and 1), with ω fixed. It is easy to see that the algorithm is nondecreasing (that is, the log likelihood ℓ never decreases) and converges, as step 1 involves ML estimation of parameters in the beta and gamma distributions, and step 2 involves comparing two points.

Note that the β–γ algorithm becomes the CoG${}_{0}$ and CoG${}_{N}$ algorithms if the beta and gamma densities are replaced by normal densities (with the same or different variances for CoG${}_{0}$ and CoG${}_{N}$, respectively).

For illustration we applied the CoG${}_{0}$ algorithm to a “thinned” sample of 1,000 points from the MCMC sample of figure 3*a* generated in the bpp analysis of the 500 noncoding *Heliconius* loci. We used three initial conditions (three rows in supplementary fig. S6, Supplementary Material online). The last plot on each row is a summary of the final processed sample. Thus the first two runs produced the same posterior, while the third run produced its mirror image.

*Algorithms CoG${}_{0}$, CoG${}_{N}$, and β–γ for the double-BDI model.* Under the double-BDI model (fig. 6*a*), eight parameters are involved in the unidentifiability, with $\Theta =(\varphi_{X},\varphi_{Y},\theta_{X},\theta_{Y},\varphi_{Z},\varphi_{W},\theta_{Z},\theta_{W})$. There are four within-model unidentifiable towers, so that $z_{t}$ takes four values (0,1,2,3), as follows (eq. 3)

-
$z_{t}=0$ : if the parameters are in $\Theta_{1}$, do nothing.
-
$z_{t}=1$ : if in $\Theta_{2}$, let $\varphi_{Z}=1-\varphi_{Z}$, $\varphi_{W}=1-\varphi_{W}$, and swap $\theta_{Z}$ and $\theta_{W}$.
-
$z_{t}=2$ : if in $\Theta_{3}$, let $\varphi_{X}=1-\varphi_{X}$, $\varphi_{Y}=1-\varphi_{Y}$, swap $\theta_{X}$ and $\theta_{Y}$, swap $\varphi_{Z}$ and $\varphi_{W}$, and swap $\theta_{Z}$ and $\theta_{W}$;
-
$z_{t}=3$ : if in $\Theta_{4}$, let $\varphi_{X}=1-\varphi_{X}$, $\varphi_{Y}=1-\varphi_{Y}$, swap $\theta_{X}$ and $\theta_{Y}$, and let $\varphi_{Z}=1-\varphi_{W}$ and $\varphi_{W}=1-\varphi_{Z}$.

We use the same strategy as in the BDI model and implement the three algorithms (CoG${}_{0}$, CoG${}_{N}$, and β–γ) as before. For β–γ, we fit four beta distributions to φs and four gamma distributions to θs, with 16 parameters in ω. We prefer the tower in which the introgression probabilities are small and initialize the algorithm accordingly. The algorithm similarly loops through two steps. In step 1, we calculate the center of gravity (represented by the means) or estimate parameters $\hat{\omega }$ to fit the beta and gamma densities, with the transforms *z* fixed. For CoG${}_{0}$ and CoG${}_{N}$, this step involves calculating the sample means and variances for the eight parameters in Θ, while for β–γ, it involves a 16-D optimization problem (or eight 2-D optimization problems) for fitting the beta and gamma distributions by ML. In step 2, we compare the four positions for each sample point when the center of gravity or parameters $\hat{\omega }$ are fixed.

*Implementation.* To apply the rule and the algorithms developed here, we need to identify the BDI event and the parameters involved in the unidentifiability, that is, ($\varphi_{X},\varphi_{Y},\theta_{X},\theta_{Y}$) under the BDI model, or ($\varphi_{X},\varphi_{Y},\theta_{X},\theta_{Y},\varphi_{Z},\varphi_{W},\theta_{Z},\theta_{W}$) under double-BDI. The algorithm is then used to process the MCMC sample. If there are multiple BDI or double-BDI events between sister species, one may simply apply the postprocessing algorithm multiple times. For instance, three rounds of postprocessing may be applied for the model of figure 5*a* (for the BDI events between *A* and *B*, between *D* and *E*, and between *S* and *U*, respectively), while the model of 5*b* requires two rounds (for the BDI between *D* and *E*, and between *S* and *U*).

The algorithms are implemented in C and require minimal computation and storage. Processing $5\times 10^{5}$ samples takes several rounds of iteration and a few seconds of running time, mostly spent on reading and writing files. The algorithms are integrated into the bpp program (Flouri et al., 2018) so that MCMC samples from various BDI models are postprocessed and summarized automatically. We also provide a stand-alone program in the github repository abacus-gene/bpp-msci-D-process-mcmc/.

## Results

### Introgression between *Heliconius melpomene* and *H. timareta*

We fitted the BDI model of figure 2 to the genomic sequence data from three species of *Heliconius* butterflies: *H. melpomene*, *H. timareta*, and *H. numata* (Edelman et al., 2019; Thawornwattana et al., 2022). When we used the first 500 loci, either noncoding or exonic, there was substantial uncertainty in the posterior of $\varphi_{X}$ and $\varphi_{Y}$, and the MCMC jumped between the twin towers, and the marginal posteriors had two modes, due to label switching (fig. 3*a* and supplementary figure S1*a*, Supplementary Material online). Postprocessing of the MCMC sample using the new algorithms led to single-moded marginal posterior distributions (fig. 3*b–d* and supplementary fig. S1*b*–*d*, Supplementary Material online). The three algorithms produced extremely similar results for both datasets. For example, the posterior mean and 95% CI for $\varphi_{X}$ from the noncoding data were 0.356 (0.026, 0.671) by CoG${}_{0}$, 0.357 (0.026, 0.674) by CoG${}_{N}$, and 0.354 (0.022, 0.664) by β–γ, while those for $\varphi_{Y}$ were 0.103 (0.000, 0.304) by CoG${}_{0}$ and CoG${}_{N}$, and 0.104 (0.000, 0.306) by β–γ.

We then analyzed all the noncoding and exonic loci on chromosome 1, and then all the autosomal loci (table 1). With the large datasets, the parameters were better estimated with narrower CIs and the unidentifiable towers were well separated. In fact, the MCMC visited only one of the two towers, but the visited tower was well explored so that multiple runs produced highly consistent results after label-switching was removed using the relabeling algorithms. When we started the MCMC with small values for $\varphi_{X}$ and $\varphi_{Y}$, postprocessing of the MCMC samples often had no effect.

Estimates of parameters from all six datasets are summarized in table 1. The introgression probabilities had overlapping CIs in datasets of different sizes, but $\varphi_{X}$ was smaller in the larger datasets, with posterior means and 95% highest-probability-density (HPD) CIs for the noncoding data to be 0.354 (0.022, 0.664) at L=500, 0.124 (0.007, 0.243) for chromosome 1, and 0.036 (0.001, 0.064) for all autosomal loci. Results for the exonic loci showed the same pattern. The rate appeared to be higher for chromosome 1 than the rest of the autosome. Introgression probability $\varphi_{Y}$ was more similar among the datasets, at about ∼10%. We note that φ in the MSci model reflects the long-term effects of gene flow and selection purging introgressed alleles, influenced by linkage to gene loci under natural selection (Martin and Jiggins, 2017). As a result, the introgression rates are expected to vary across the chromosome or genome. It will be interesting to analyze larger datasets with more samples per species to examine the variation in the rate of gene flow across the genome.

Note that *H. melpomene* has a widespread geographical distribution whereas *H. timareta* is restricted to the Eastern Andes. The small $\theta_{M}$ estimates are most likely due to the fact that the *H. melpomene* sample was from a partially inbred strain to avoid difficulties with genome assembly. Estimates of θs and τs were smaller for the coding loci than for the noncoding loci, presumably due to purifying selection removing deleterious nonsynonymous mutations (Shi and Yang, 2018).

### Analysis of Data Simulated under the Double-BDI Model of figure 6*a*

We conducted a small simulation to illustrate the feasibility of the double-BDI model (fig. 6), simulating 10 replicate datasets of L=500, 2,000, and 8,000 loci. The three algorithms were used to process the MCMC samples, before they were summarized.

**Fig. 7.**
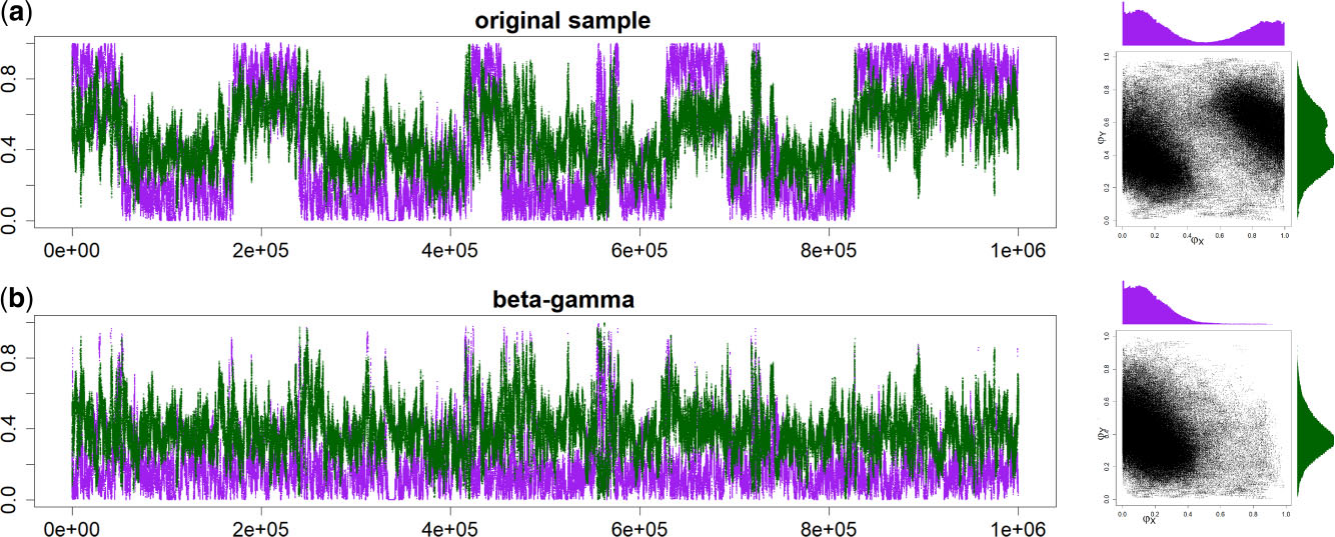
Trace plots of MCMC samples and 2-D scatter plots for parameters $\varphi_{Z}$ (purple) and $\varphi_{W}$ (green) (*a*) before and (*b*) after the postprocessing of the MCMC samples for the double-BDI model of figure 6*a*. Postprocessing used the β–γ algorithm (*b*), while CoG${}_{N}$ and CoG${}_{0}$ produced nearly identical results (not shown). This is for replicate 2 for L=500 loci (see fig. 8).

**Fig. 8.**
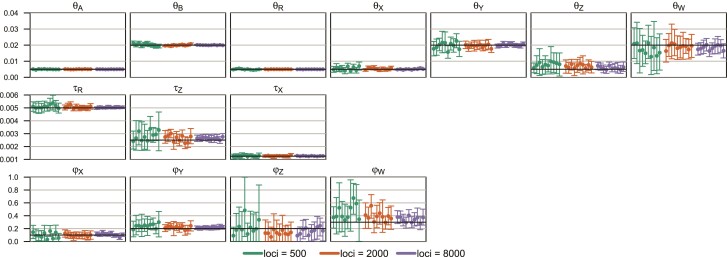
Posterior means and the 95% HPD CIs in 10 replicate datasets of L=500, 2,000, and 8,000 loci, simulated and analyzed under the double-BDI model of figure 6*a*. The MCMC samples are postprocessed using the β–γ algorithm before they are summarized (e.g. fig. 7). Eight parameters are involved in the label-switching unidentifiability: $\varphi_{X}$, $\varphi_{Y}$, $\theta_{X}$, $\theta_{Y}$, $\varphi_{Z}$, $\varphi_{W}$, $\theta_{Z}$, and $\theta_{W}$ (see fig. 6).

For the case of L=500, a typical case is shown in figure 7. While there are four unidentifiable towers in the 8-D posterior space (eq. 3), the MCMC visited only two of them, with different values for parameters around the BDI event at the node *ZW*. The dataset of L=500 loci are very informative about the parameters for the BDI event at node *XY* ($\varphi_{X}$, $\varphi_{Y}$, $\theta_{X}$, $\theta_{Y}$), so that these had highly concentrated posteriors with well-separated towers. We started the Markov chains with small values (e.g., 0.1 and 0.2) for $\varphi_{X}$ and $\varphi_{Y}$, so that the sampled points were all around the correct tower for those parameters. If the chain started with large $\varphi_{X}$ and $\varphi_{Y}$, it would visit a “mirror” tower. Thus, postprocessing of the MCMC samples mostly affected parameters around the BDI event at *ZW* ($\varphi_{Z}$, $\varphi_{W}$, $\theta_{Z}$, $\theta_{W}$). Figure 7 shows the effects on parameters $\varphi_{Z}$ and $\varphi_{W}$ using the β–γ algorithm. The CoG${}_{0}$ and CoG${}_{N}$ algorithms produced nearly identical results, and all algorithms were effective in removing label switching. The postprocessed samples were summarized to calculate the posterior means and the HPD CIs (fig. 8).

At L=2,000 or 8,000 loci, the four towers were well separated and the MCMC visited only one of them. Applying the postprocessing algorithms either had no effect or, in rare occasions, moved all sampled points from one tower to another.

Posterior means and the 95% HPD CIs for all parameters were summarized in figure 8. Parameters around the BDI event at *ZW* ($\varphi_{Z}$, $\varphi_{W}$, $\theta_{Z}$, $\theta_{W}$) are the most difficult to estimate. Nevertheless, the CIs for all parameters were smaller at L=8,000 than at L=500 or 2,000, and the posterior means were converging to the true values. Note that while the simulation was conducted using one set of correct parameter values (say, $\Theta_{1}$ of fig. 6), we considered the estimates to be good if they were close to any of the four unidentifiable towers (say, $\Theta_{2}$, $\Theta_{3}$, or $\Theta_{4}$). This is analogous to treating the estimate as correct in Bayesian clustering if the true model includes two groups in proportions $p_{1}=10\text{\%}$ and $p_{2}=90\text{\%}$ with means $\mu_{1}=100$ and $\mu_{2}=1$, while the method of analysis infers two groups in proportions $p_{1}^{\prime }=90\text{\%}$ and $p_{2}^{\prime }=10\text{\%}$ with means $\mu_{1}^{\prime }=1$ and $\mu_{2}^{\prime }=100$. Just as $\Theta =(p_{1},\mu_{1},\mu_{2})$ and $\Theta^{\prime }=(p_{2},\mu_{2},\mu_{1})$ are unidentifiable towers and equally correct answers in the clustering problem, here $\Theta_{1}$, $\Theta_{2}$, $\Theta_{3}$, and $\Theta_{4}$ are equally correct answers.

### Analysis of Data Simulated with One BDI Event with Poorly Separated Modes

We simulated a challenging dataset for the relabeling algorithms, with L=500 loci, under the BDI model of figure 1*a* with $\left(\varphi_{X},\varphi_{Y})=(0.7,0.2\right)$ (see supplementary table S1, Supplementary Material online). As $\varphi_{X}$ and $\varphi_{Y}$ were not too far away from $\frac{1}{2}$ and the dataset was small, the posterior modes were poorly separated, with considerable mass near $\left(\frac{1}{2},\frac{1}{2}\right)$. In the unprocessed MCMC sample, $\varphi_{X}$ had two modes around 0.8 and 0.2 and the chain was switching between them (supplementary fig. S7*a*, Supplementary Material online). The posterior means were at 0.51 for $\varphi_{X}$ and 0.50 for $\varphi_{Y}$, close to $\frac{1}{2}$ (supplementary fig. S7*a*, Supplementary Material online). These are misleading summaries, as the sample was affected by label switching. In the processed samples (supplementary fig. S7*b*–*d*, Supplementary Material online), label switching was successfully removed and both $\varphi_{X}$ and $\varphi_{Y}$ were single-moded. The three algorithms (β–γ, CoG${}_{N}$, and CoG${}_{0}$) produced similar results, with single-moded posterior, around the tower $\left(\varphi_{X},\varphi_{Y})=(0.7,0.2\right)$. The posterior means of $\left(\varphi_{X},\varphi_{Y}\right)$ were (0.755, 0.447), (0.766, 0.461), and (0.765, 0.462) for the three algorithms, β–γ, CoG${}_{N}$, and CoG${}_{0}$, respectively (supplementary table S1, Supplementary Material online). The estimates from β–γ were slightly closer to the true values than those from CoG${}_{N}$ and CoG${}_{0}$. The three relabeling algorithms worked well even when the posterior modes were poorly separated.

Parameters not involved in label-switching, such as the species divergence and introgression times ($\tau_{R},\tau_{X}$) and the population sizes for the modern species and for the root ($\theta_{A},\theta_{B},\theta_{R}$), were well estimated, with the posterior means close to the true values and with narrow CIs (supplementary table S1, Supplementary Material online). However, parameters involved in label switching ($\varphi_{X},\varphi_{Y},\theta_{X},\theta_{Y}$) were poorly estimated at this data size (with L=500 loci), because of the difficulty to separate the two towers and the influence of the priors. The estimates should improve if more loci are used in the data. To confirm this expectation, we simulated two more datasets with L=2,000 and 8,000 loci, respectively. In those two datasets, parameters not involved in label switching ($\tau_{R},\tau_{X},\theta_{A},\theta_{B},\theta_{R}$) had very narrow CIs (supplementary table S1, Supplementary Material online). At L=8,000, the posterior means of $\Theta =(\varphi_{X},\varphi_{Y})$ were closer to the true values (0.7, 0.2) and the 95% CIs were narrower than in the small dataset of L=500 (supplementary table S1, Supplementary Material online). Note that ancestral population sizes (such as $\theta_{X}$ and $\theta_{Y}$) are hard to estimate even in models of unidirectional introgression which do not have label-switching issues (Huang et al., 2020).

## Discussion

### Data Size, Precision of Parameter Estimation, MCMC Convergence, and the Utility of the Relabeling Algorithms

We have observed three kinds of behaviors of the MCMC algorithm and the relabeling algorithms depending on the data size. In small datasets, the parameters are poorly estimated with large uncertainties, and the posterior modes (the unidentifiable towers) are not well separated. In such cases, applying simple constraints (such as $\varphi_{X}<\frac{1}{2}$) is problematic because the truncation distorts the marginal summaries of the posterior, with different constraints producing different posterior summaries (Richardson and Green, 1997; Celeux et al., 2000; Stephens, 2000). The relabeling algorithms are preferable. An example is the small dataset of L=500 loci simulated under the model of one BDI event (supplementary fig. S7 and table S1, Supplementary Material online).

In intermediate datasets, the parameters are well estimated, the posterior modes are well separated, but the MCMC algorithm jumps between the modes, switching labels. In such cases, any of the relabeling algorithms will work well. If the posterior modes are far away from the boundary defined by the constraints (such as $\varphi_{X}<\frac{1}{2}$), even imposing simple constraints will work as well. Examples include the two small butterfly datasets with L=500 loci (fig. 3 and supplementary fig. S1, Supplementary Material online), and the datasets simulated under the double-BDI model (fig. 7).

Finally, in very large datasets, the parameters are extremely well estimated with very narrow CIs, and the posterior modes are so sharply concentrated that the MCMC algorithm stays on one of the unidentifiable towers and never moves to the mirror towers. Furthermore, in multiple runs of the same analysis the MCMC may be “stuck” on different towers. In such cases, the relabeling algorithms will either not move any sample points at all or move all points from one tower to another, and any of the algorithms will work well. This scenario is common in analyses of large genomic datasets with thousands of loci, such as the large noncoding and exonic datasets from the *Heliconius* butterflies (fig. 2); See Finger et al. (2022) and Thawornwattana et al. (2022) for many more examples.

We note that in all three scenarios, the relabeling algorithms (in particular, the β–γ algorithm) were either better or not worse than the alternatives such as imposing simple constraints. Given that even the β–γ algorithm involves minimal computation, we recommend its automatic use in all cases. Samples from different runs visiting different unidentifiable modes may be merged before postprocessing using the relabeling algorithm.

In theory, if the MCMC has converged and is mixing well and the algorithm is run long enough, it should visit the unidentifiable towers with exactly the same probability and the means of introgression probabilities from the unprocessed samples should be $\frac{1}{2}$. One might expect this to provide a useful criterion for diagnosing the convergence of MCMC algorithms. Indeed Jasra et al. (2005) regarded it “a *minimum* requirement of convergence for a mixture posterior to be such that we have explored all possible labellings of the parameters.” Here the labelings correspond to the unidentifiable towers. We suggest that this requirement is too stringent and unnecessary. As discussed above, in large genomic datasets, the posterior may be highly concentrated, and the chain may never jump between the towers even in very long MCMC runs. While the chain may be visiting different mirror towers in different runs of the same analysis, each chain may be exploring the space around the visited tower thoroughly, and after label switching is removed, the MCMC samples from the different runs may produce nearly identical posterior summaries, suggesting that reliable inference is possible. In simulations of large datasets, the posterior estimates after label-switching problems are removed converge to the true values (e.g., Flouri et al., 2020, fig. S10A). One could include a random permutation step in each MCMC iteration, so that the unidentifiable towers are visited with equal probabilities, but this does not eliminate the need for postprocessing the MCMC sample to remove label switching. We suggest that exploration of all unidentifiable towers is unnecessary for correct inference and should not be used as a criterion for diagnosing MCMC convergence. Instead convergence diagnosis should be applied after the MCMC sample is processed to remove label switching. For example, one should run the same analysis multiple times and confirm that the posterior summaries when the MCMC samples are processed and mapped onto the same tower are consistent between runs. The efficiency of the MCMC algorithm or the effective sample size (ESS) (Yang and Rodríguez, 2013) should also be calculated using the processed samples.

### Identifiability of MSci Models with Unidirectional Introgressions

**Fig. 9.**
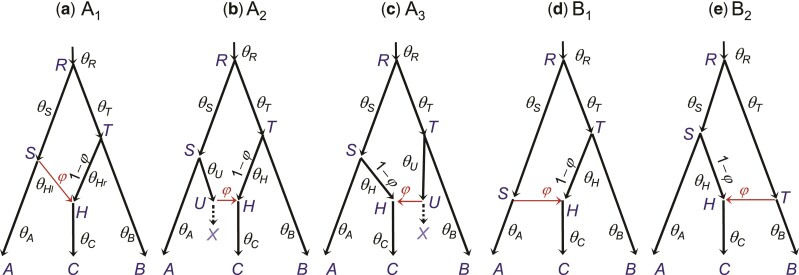
Species trees for three species (*A*, *B*, and *C*) illustrating MSci models of types A and B defined by Flouri et al. (2020, figure 1). (*a*–*c*) Three interpretations of MSci model A (Flouri et al., 2020, figure 1) are indistinguishable/unidentifiable. (*d*,*e*) Two versions of MSci model B (Flouri et al., 2020, figure 1) are identifiable.

The identifiability of MSci models involving unidirectional introgression (UDI) events appears to be simpler than for BDI models (Flouri et al., 2020; Jiao et al., 2021). MSci model A (Flouri et al., 2020, figure 1) is consistent with three different biological scenarios (fig. 9*a–c*). In scenario A${}_{1}$, two species *SH* and *TH* merge to form a hybrid species *HC*, but the two parental species become extinct after the merge. This scenario may be rare. In scenario A${}_{2}$, species *SUX* contributes migrants to species *THC* at time $\tau_{H}$ and has since become extinct or is unsampled in the data. In scenario A${}_{3}$, *TUX* is the extinct or unsampled ghost species. The three scenarios are unidentifiable using genomic data. Model B${}_{1}$ assumes introgression from species *RA* to *TC* at time $\tau_{S}=\tau_{H}$ (fig. 9*d*). This is distinguishable using genetic data from the alternative model B${}_{2}$ in which there is introgression from *RB* to *SC* (fig. 9*e*). Note that models B${}_{1}$ and B${}_{2}$ are both special cases of model A${}_{1}$ with different constraints (that is, $\tau_{S}=\tau_{H}<\tau_{T}$ for model B${}_{1}$ and $\tau_{S}>\tau_{H}=\tau_{T}$ for model B${}_{2}$).

Note that the sampling configuration may affect the identifiability of parameters in the model (Yu et al., 2012; Zhu and Degnan, 2017). The simplest such example may be the population size parameter (θ). If at most one sequence per locus is sampled from a species, the population size for that species will be unidentifiable. Similarly, if no more than one sequence per locus can enter an ancestral population when we trace the genealogy of the sampled sequences backwards in time, θ for that ancestral species will be unidentifiable. Such unidentifiability disappears when multiple sequences per species are sampled. Note that a diploid sequence is equivalent to two haploid sequences. Similarly introgression models that are unidentifiable with one sampled sequence per species may become identifiable when multiple sequences per species are sampled (Zhu and Degnan, 2017).

**Fig. 10.**
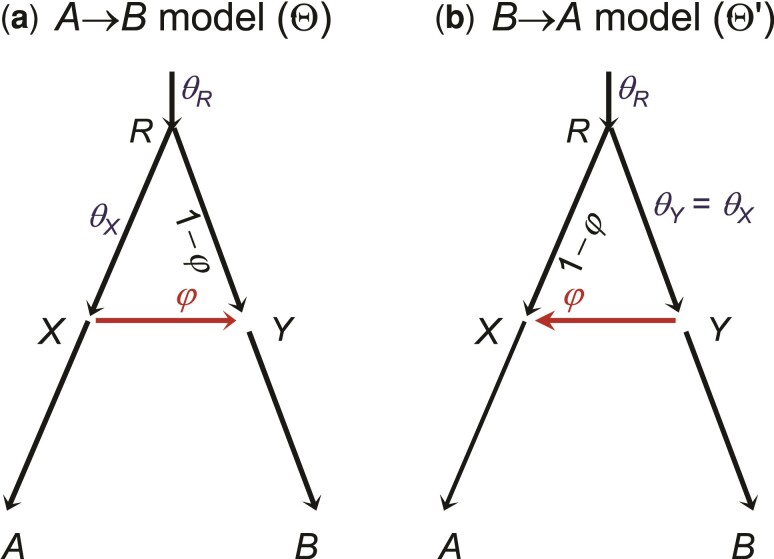
The unidirectional introgression model for two species, given multilocus sequence data with one sequence per species per locus, is unidentifiable, with parameter mappings $\Theta =(\tau_{R},\tau_{X},\theta_{X},\theta_{R},\varphi_{Y})$ in (*a*) and $\Theta^{\prime }=(\tau_{R},\tau_{X},\theta_{Y},\theta_{R},\varphi_{X})$ in (*b*). Note that with one sequence per species, $\theta_{A},\theta_{B},\theta_{Y}$ in the A→B model are unidentifiable, as are $\theta_{A},\theta_{B},\theta_{X}$ in the B→A model. If multiple sequences are available per species per locus, all parameters are identifiable and the two models with gene flow in different directions are identifiable as well.

An interesting example concerns the UDI model in the case of two species with one sequence sampled per species per locus, which creates a cross-model unidentifiability (fig. 10*a* and *b*). In both the A→B and B→A introgression models, five parameters are estimable, but the two models are unidentifiable, because they produce exactly the same distribution of the coalescent time between the two sequences at any locus. In other words, with a pair of sequences per locus, one can estimate the timing and strength of introgression, but not its direction. If multiple sequences are available per species per locus, the two models are identifiable, as are the eight parameters in each model.

Even if the model is mathematically identifiable with one sequence per species per locus, including multiple samples per species (in particular, for species that are descendants of a hybridization node in the species tree) can boost the information content in the data dramatically. Thus, we recommend the use of multiple samples per species in studies of cross-species gene flow, and suggest that the most interesting scenario for studying unidentifiability of models of gene flow should be full-likelihood analysis of multilocus sequence data, with multiple sequences sampled per species.

It is noteworthy that many parameter settings and data configurations exist in which some parameters are hard to estimate, because the data lack information about them. For example, ancestral population sizes for short and deep branches in the species tree are hard to estimate, because most sequences sampled from modern species may have coalesced before reaching that population when we trace the genealogy of the sample backwards in time (Huang et al., 2020). Similarly, if few sequences reach a hybridization node, there will be little information in the data about the introgression probabilities at that node. In such cases, even if the model is identifiable mathematically, it may be nearly impossible to estimate the parameters with any precision even with large datasets.

In some cases, certain parameters may be nearly at the boundary of the parameter space, and this may create near unidentifiability with multiple modes in the posterior. For example, two speciation events that occur in rapid succession will generate a very short branch in the species tree with a near trichotomy in the species tree. Then MSci models that posit the same introgression events but different histories of species divergences will fit the data nearly equally well and become multiple modes in the posterior space (see Finger et al., 2022 for an example). Similarly introgression probabilities near 0 or 1 can also create nearly equally good explanations of the data and become multiple modes in the posterior. In such situations, the MCMC samples around different modes should be summarized separately.

### Unidentifiability of Heuristic Methods

As mentioned in Introduction, the unidentifiability discussed in this paper concerns the intrinsic nature of the inference problem when introgression models are applied to genomic sequence data, and thus applies to not only full-likelihood methods but also heuristic methods based on summaries of the sequence data. Indeed a model that is unidentifiable by a full-likelihood method must be unidentifiable by any heuristic method. In contrast, a model that is identifiable by a full-likelihood method may still be unidentifiable by a heuristic method as the heuristic method may not be using all information in the data. Here we briefly discuss a few heuristic methods, focusing on their common features. Interested readers may consult the recent reviews by Elworth et al. (2019) and Hibbins and Hahn (2021). Heuristic methods developed up to now are mostly of two kinds, based on either genome-wide averages or estimated gene trees for genomic segments (loci).

The popular *ABBA*-*BABA* test (Durand et al., 2011) uses the parsimony-informative site patterns across the genome to detect gene flow. Consider three populations/species $S_{1}$, $S_{2}$, and $S_{3}$, with the given phylogeny $\left((S_{1},S_{2}),S_{3}\right)$, plus an outgroup species *O*. There are three parsimony-informative site patterns: *ABBA*, *BABA*, and *BBAA*. Here *A* and *B* represent any two distinct nucleotides and *BBAA* means $S_{1}$ and $S_{2}$ have the same nucleotide while $S_{3}$ and *O* have another. For very closely related species, one may consider nucleotide *A* in the outgroup as the ancestral allele and *B* the derived allele. Site pattern *BBAA* matches the species tree, while *ABBA* and *BABA* are the mismatching patterns. Given the species tree with no gene flow, the two mismatching patterns have the same probability, but when there is gene flow between $S_{1}$ (or $S_{2}$) and $S_{3}$, they will have different probabilities. The difference between the two mismatching site-pattern counts can then be used to test for the presence of gene flow (Durand et al., 2011):

(9) $$D=\frac{n_{ABBA}-n_{BABA}}{n_{ABBA}+n_{BABA}}.$$

The *D*-statistic may also be seen as a comparison between the number of derived alleles shared by $S_{2}$ and $S_{3}$ with that shared by $S_{1}$ and $S_{3}$. It can test for the presence of gene flow, but provides no information about its direction, timing, or strength.

The site pattern counts can also be used to estimate the introgression probability, as in the program hyde (Blischak et al., 2018; Kubatko and Chifman, 2019):

(10) $$\hat{\varphi }=\frac{n_{BBAA}-n_{BABA}}{n_{BBAA}-2n_{BABA}+n_{ABBA}}.$$

This is based on the hybrid speciation model (assuming $\tau_{S}=\tau_{H}=\tau_{T}$ and $\theta_{S}=\theta_{T}$ in model A${}_{1}$ of fig. 9). The estimate may be biased if this symmetry assumption does not hold. Instead of the parsimony-informative site patterns, the average sequence distance between species may be similarly used to construct a test (Hahn and Hibbins, 2019). Furthermore, the *D*-statistic has been extended to the case of five species, with a symmetric species tree assumed, in the so-called $D_{\mathrm{FOIL}}$ test, with the aim to detect the direction of gene flow (Pease and Hahn, 2015).

Note that both the site-pattern counts and between-species distances are genome-wide averages. If the data consist of multilocus sequence alignments, they can be merged (concatenated) into a super-alignment to calculate those statistics. A great advantage of those methods is that they involve minimal computation. A serious drawback is that they do not make use of information in genealogical variations across the genome (Lohse and Frantz, 2014; Shi and Yang, 2018). Like the coalescent process, gene flow between species creates stochastic fluctuations in the genealogical history (gene tree topology and coalescent times) across the genome, with the probability distribution given by the parameters in the multispecies coalescent model with gene flow, including species divergence times, effective population sizes for modern and ancestral species, and the directions and rates of gene flow. As a result, there is important information about those parameters in such genomic variation, but this information is ignored by those methods. In other words, those methods use the total or *mean* site-pattern counts but fail to use information in the *variances* in the site-pattern counts among loci. As a result, most parameters in the coalescent model with introgression are unidentifiable by the heuristic methods mentioned above. None of them can detect signals of gene flow between sister species, and for nonsister species, none of them can estimate the introgression probabilities when gene flow occurs in both directions (e.g., $\varphi_{X}$ and $\varphi_{Y}$ in fig. 1*a* or α and β in supplementary fig. S3*a*, Supplementary Material online).

The second kind of heuristic methods use reconstructed gene tree topologies for multiple loci as the input data. Consider again the species quartet $S_{1}$, $S_{2}$, $S_{3}$, and *O* (outgroup), with the given phylogeny $\left((S_{1},S_{2}),S_{3}\right)$, and one sampled sequence per species. The two mismatching gene trees $\left((S_{2},S_{3}),S_{1}\right)$ and $\left((S_{3},S_{1}),S_{2}\right)$ have the same probability if there is coalescence but no gene flow, but different probabilities if there is in addition gene flow between the nonsister species (between $S_{1}$ and $S_{3}$ or between $S_{2}$ and $S_{3}$). Thus the frequencies of gene tree topologies can be used to estimate the introgression probability, as in the snaq method (Solis-Lemus and Ane, 2016, see also Yu et al., 2012). As there are only two free quantities (frequencies of three gene trees with the sum to be 1), the approach can estimate the internal branch length in coalescent units and the introgression probability, but not any other parameters in the model.

In the general case, the probabilities of gene tree topologies under any introgression model can be calculated by summing over the compatible coalescent histories (Yu et al., 2012, 2014). The probability distribution of gene tree topologies can then be used to distinguish among different introgression models and to estimate the parameters in the introgression model by ML (as in PhyloNet; Wen et al., 2018), treating gene tree topologies as data. A concern with the two-step method is that the estimated gene trees may involve uncertainties or errors, in particular when the species are closely related. Including gene tree branch lengths (coalescent times) makes many introgression models that are unidentifiable based on gene tree topologies alone become identifiable (Yu et al., 2012; Zhu and Degnan, 2017). However, two-step methods that make use of estimated branch lengths was found to perform poorly as the large uncertainties and errors in the estimated branch lengths can have a major impact on inference of species divergence and cross-species gene flow (Degnan, 2018).

There is currently a wide gap between likelihood and heuristic methods. Heuristic methods are computationally orders-of-magnitude faster than likelihood methods, which in particular do not scale well for large genomic datasets. The statistical properties of heuristic methods are also incomparably poorer than those of likelihood methods: heuristic methods are simply unable to provide any estimates for many fundamental population parameters for characterizing the evolutionary history of the species, such as the species divergence/introgression times and the population sizes of extant and extinct species. There is an acute need for improving the statistical performance of the heuristic methods and the computational efficiency of the full-likelihood methods.

Given the limitations of the heuristic methods, one should apply caution when using them to draw biological conclusions concerning gene flow between species. For example, does gene flow occur more often between sister species or between nonsister species? When gene flow occurs between two species, does it often involve one direction (UDI) or both directions (BDI)? Most heuristic methods cannot identify or detect gene flow between sister species or gene flow in both directions, but it may be erroneous to conclude that such gene flow never occurs in nature. Whether BDI or UDI is more common is an interesting empirical question, but both models provide important biological hypotheses testable using genomic sequence data. In a recent analysis of genomic sequence data from the North-American chipmunks (*Tamias quadrivittatus*), the use of the *D*-statistic and hyde detected no evidence of gene flow affecting the nuclear genome despite widespread mitochondrial gene flow (Sarver et al., 2021). However, a reanalysis of the same data using bpp revealed robust evidence for multiple ancient introgression events, involving both sister and nonsister species (Ji et al., 2021).

### Displayed Species Trees and Identifiability of MSci Models

**Fig. 11.**
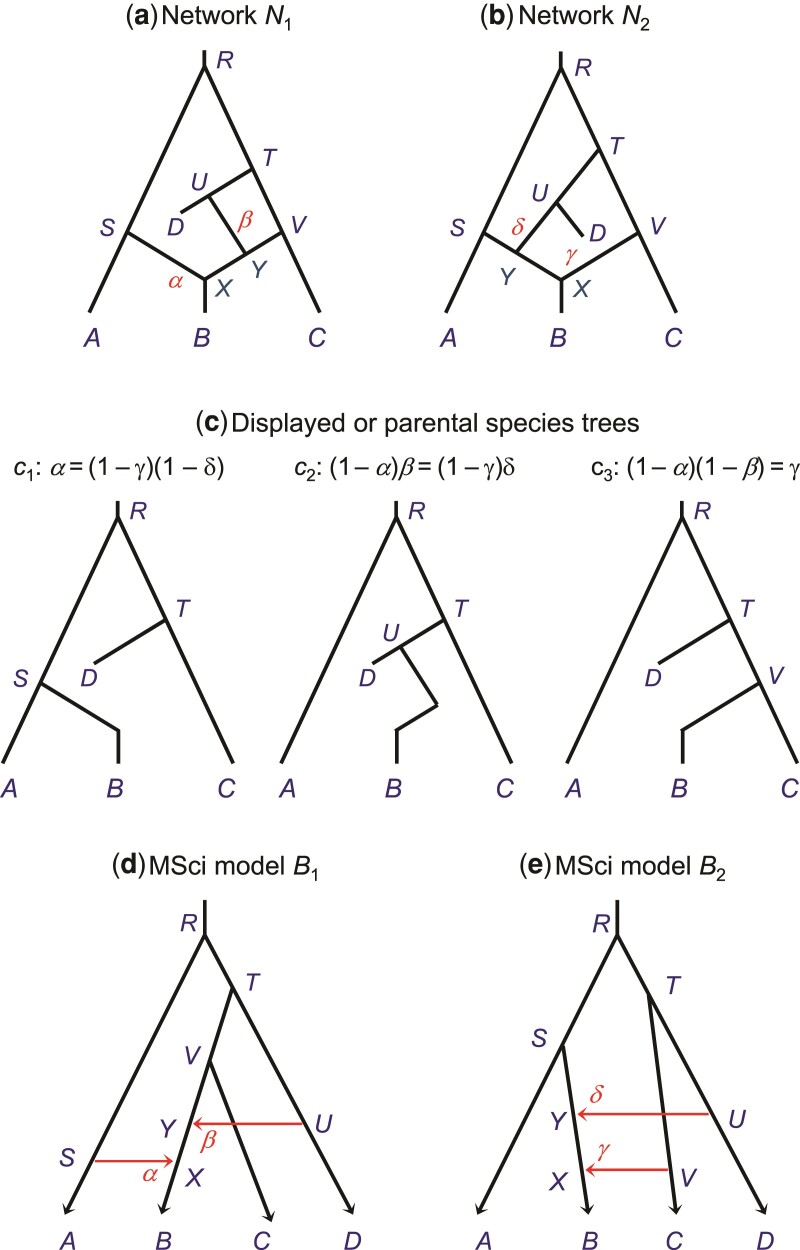
(*a*,*b*) Two phylogenetic networks for four species (A,B,C,D), each with two hybridization events from Pardi and Scornavacca (2015) that are unidentifiable using gene tree topologies with one sequence sampled per species. (*c*) Network $N_{1}$ gives rise to three ‘displayed species trees’ in probabilities α,(1−α)β, and (1−α)(1−β), while $N_{2}$ gives rise to the same three displayed species trees with probabilities (1−γ)(1−δ),(1−γ)δ, and γ. The two networks thus give the same distribution of gene tree topologies, and are thus unidentifiable. However, $N_{1}$ and $N_{2}$ are identifiable when multiple samples are taken from species *B*. (*d*,*e*) MSci models corresponding to networks $N_{1}$ and $N_{2}$. With information from branch lengths (coalescent times) and using multilocus sequence data, those models are identifiable by full-likelihood method, as are the 18 parameters in each model, including five species divergence/introgression times (τs), 11 population sizes (θs), and two introgression probabilities.

Pardi and Scornavacca (2015) studied the unidentifiability of network models using data of gene tree topologies “displayed” by the network (fig. 11). Binary species trees generated by taking different parental paths at hybridization nodes are called “displayed species trees” (Pardi and Scornavacca, 2015) or “parental species trees” (Kubatko, 2009). For example, the two network models $N_{1}$ and $N_{2}$ of figure 11*a* are unidentifiable when only one sequence is sampled per species because they induce the same three displayed species trees with the same branch lengths (Pardi and Scornavacca, 2015). However, as pointed out by Zhu and Degnan (2017), $N_{1}$ and $N_{2}$ are identifiable using gene tree topologies if multiple sequences are sampled from *B*.

Previously Kubatko (2009, eq. 3; see also Meng and Kubatko, 2009) formulated the probability distribution of gene trees (topology alone or topology with coalescent times) as a mixture over the displayed species trees. To simulate gene trees or sequence data at a locus, one samples a displayed species tree first and then simulates the gene tree and sequence alignment according to the simple MSC model (Gerard et al., 2011). This formulation is in general incorrect as it forces all sequences at the locus to take the same parental path at each hybridization node, whereas correctly there should be a binomial sampling process when two or more sequences reach a hybridization node. In model $N_{1}$ of figure 11*a*, when multiple *B* sequences reach species *X*, it should be possible for some sequences to take the left parental path while the others take the right path. The formulation is correct in the special case where each hybridization node on the species tree has at most one sequence from all its descendant populations (Zhu and Degnan, 2017).

Even though the notion that gene trees are displayed by a phylogenetic network has played a central role in many studies that attempt to use gene tree topologies to construct the phylogenetic network, examination of the displayed gene trees is not a reliable approach to studying the unidentifiability of phylogenetic network models (Zhu and Degnan, 2017). The most probable gene tree may even have a topology that is different from all of the displayed trees (Zhu and Degnan, 2017). Note that both MSci models corresponding to networks $N_{1}$ and $N_{2}$ are identifiable when genomic sequence data with multiple samples per species are analyzed using full-likelihood methods (fig. 11*d* and *e*), as are all parameters in each models (fig. 11*a*' and *b*'). In summary, we suggest that the idea of displayed species trees may not be a very useful one either for calculating the density of gene trees or for studying the identifiability of MSci models when there are multiple samples per species in the data. Instead, one should explicitly treat the biological process of coalescent and introgression in the model (Zhu and Degnan, 2017). We suggest that multiple sequences be sampled per species (in particular from species involved in hybridization or from descendant species of hybridization nodes) when genomic data are used to infer gene flow.

### Estimation of Introgression Probabilities Despite Unidentifiability

The three relabeling algorithms for postprocessing MCMC samples under the BDI model produced very similar results in the applications in this study. In particular, the simple center-of-gravity algorithms produced results that appear to be as good as the more elaborate β–γ algorithm, despite the fact that the normal distribution is a poor approximation to the posterior of introgression probabilities ($\varphi_{X}$ and $\varphi_{Y}$). This is due to the fact that the distributions (or the distances in the CoG algorithms) are used to compare the unidentifiable mirror positions of sample points only, but are not used to approximate the posterior distribution of those parameters, which are estimated by using the processed samples. For the same reasons, if there exist multiple modes in the posterior that are not due to label switching, such genuine multimodality will not be removed by the relabeling algorithms (Stephens, 2000). Similarly, while we fit independent distributions for parameters in the algorithms (eq. 6), there is no need to assume independence in the posterior for the algorithms to work.

A model with a label-switching type of unidentifiability is still useful for real data analysis. In the clustering problem, the Bayesian analysis may reveal the existence of two groups, in proportions $p_{1}$ and $p_{2}=1-p_{1}$ with means $\mu_{1}$ and $\mu_{2}$, and it does not matter if it cannot decide which group should be called “group 1.” The twin towers Θ and $\Theta^{\prime }$ under the BDI model (fig. 1) constitute a mathematically similar label-switching problem. However, Θ and $\Theta^{\prime }$ under BDI may represent different biological scenarios or hypotheses. Suppose that species *A* and *B* are distributed in different habitats (dry for *A* and wet for *B*, say), and suppose the ecological conditions have changed little throughout the history of the species. Θ with $\varphi_{X}<\frac{1}{2}$ and $\varphi_{Y}<\frac{1}{2}$ may mean that species *A* has been in the dry habitat over the whole time period since species divergence at time $\tau_{R}$, while species *B* has been in the wet habitat, and they came into contact and exchanged migrants at time $\tau_{X}$. In contrast, $\Theta^{\prime }$ with $\varphi_{X}^{\prime }>\frac{1}{2}$ and $\varphi_{Y}^{\prime }>\frac{1}{2}$ may mean that species *A* was in the wet habitat and species *B* was in the dry habitat since species divergence at time $\tau_{R}$, but when they came into contact at time $\tau_{X}$ they nearly replaced each other, switching places, so that today species *A* is found in the dry habitat while *B* in the wet habitat. The two sets of parameters Θ and $\Theta^{\prime }$ may thus mean different biological hypotheses. As genomic data from modern species provide information about the order and timings of species divergences and cross-species introgressions, but not about the geographical locations and ecological conditions in which the divergences and introgressions occurred, such biological scenarios are unidentifiable using genomic data and become unidentifiable towers in the posterior distribution in Bayesian analysis of genomic data under the MSci model. Unidentifiable models discussed in this paper are all of this nature. The algorithms we developed in this paper remove label switching in the MCMC sample, but do not remove the unidentifiability of the BDI models. The researcher has to be aware of the unidentifiability and use external information (such as fossil evidence or ancient climate data) to choose between such equally supported explanations of the genomic data.

In the above example, the scenario of near-complete replacement represented by $\Theta^{\prime }$ may be implausible and the model with small introgression probabilities may be preferable for most systems. In our relabeling algorithms, we start with small $\varphi_{X}$ and $\varphi_{Y}$ as much as possible (through the initial condition $\varphi_{X}+\varphi_{Y}<1$). When the introgression probabilities are intermediate, the biological interpretations may not be so clear-cut, but unidentifiability exists nevertheless. In the example of supplementary figure S7 and table S1, Supplementary Material online for the simulated data with one BDI event, the choice between the two unidentifiable towers $\Theta =(\varphi_{X},\varphi_{Y})=(0.7,0.2)$ and $\Theta^{\prime }=(0.3,0.8)$ may not be easy.

Another strategy may be to modify the BDI model so that it becomes identifiable. In the current implementation in bpp, each branch in the species tree is assigned its own population size parameter (Flouri et al., 2020). We note that if all species on the species tree are assumed to have the same population size (θ), unidentifiability persists. However, if we assume that the population size remains unchanged by the introgression event: for example, $\theta_{X}=\theta_{A}$ and $\theta_{Y}=\theta_{B}$ in figure 1, the model becomes identifiable. The assumption of the same population size before and after an introgression event appears to be plausible biologically. It reduces the number of parameters by two for each BDI event, and removes unidentifiability. It may be worthwhile to implement such models.

## Methods and Materials

### Introgression in *Heliconius* Butterflies

We fitted the BDI model to the genomic sequence data for three species of *Heliconius* butterflies: *H. melpomene*, *H. timareta*, and *H. numata* (Consortium, 2012; Martin et al., 2013). The species tree or MSci model assumed is shown in figure 2, with introgression between *H. melpomene* and *H. timareta*. The two species are known to hybridize, although no attempt has yet been made to infer the direction or strength of introgression (except for colour-pattern genes; Martin et al., 2013). There are 31,166 autosomal noncoding loci and 36,138 autosomal exonic loci, with one diploid sequence sampled per species per locus. The sequence length ranges from 11 to 991 bps (median 93) for the noncoding loci and from 11 to 10,672 bps (median 112) for the exonic loci. The data were prepared using the same procedure and filters as in Thawornwattana et al. (2022). We analyzed six datasets under the same model, with the noncoding and exonic loci in separate datasets: the first 500 loci on chromosome 1, all loci on chromosome 1 (2,592 noncoding or 3,023 exonic loci), and all autosomal loci (table 1).

Note that a diploid sequence from each species is equivalent to two haploid sequences, so that the population size parameter (θ) for that species is estimable. Heterozygotes in the diploid sequence are represented by IUPAC ambiguity codes (e.g., with Y meaning a T/C heterozygote) and resolved into compatible nucleotides in bpp using an analytical integration algorithm (Gronau et al., 2011; Flouri et al., 2018), which averages over all possible genotypic phase resolutions of heterozygote sites, weighting them according to their likelihood based on the sequence alignment at the locus. In simulations, this approach had indistinguishable performance from analysis of fully and correctly phased genomic sequences (Gronau et al., 2011; Huang et al., 2021).

We used gamma priors for the population sizes (θ) and for the age of the root ($\tau_{0}$): θ∼G(2,400) with the mean 0.005 substitution per site, and τ∼G(2,400) with mean 0.005. The introgression probabilities were assigned beta priors $\varphi_{X},\varphi_{Y}∼B(1,1)$, which is the uniform U(0,1). We used a burn-in of 16,000 iterations, and then took $2\times 10^{5}$ samples, sampling every five iterations. Running time on a server using nine threads of Intel Xeon Gold 6154 CPU (3.0GHz) was about one hour for the small datasets or L=500 loci, ∼10 h for the datasets of chromosome 1, and ∼4 days for the datasets of all autosomal loci.

Convergence of the MCMC algorithms was assessed by checking for consistency between independent runs, taking into account possible label-switching issues.

### Simulation under the Double-BDI Model

We simulated and analyzed data under the double-BDI model of figure 6. Gene trees with branch lengths (coalescent times) were simulated under the MSci model and given the gene trees, sequences were evolved along the branches on the gene tree under the JC model (Jukes and Cantor, 1969). The parameters used were $\varphi_{X}=0.1$, $\varphi_{Y}=0.2$, $\varphi_{Z}=0.2$, $\varphi_{W}=0.3$, $\tau_{R}=0.005$, $\tau_{Z}=\tau_{W}=0.0025$, $\tau_{X}=\tau_{Y}=0.00125$, $\theta_{R}=\theta_{Z}=\theta_{X}=\theta_{A}=0.005$, and $\theta_{W}=\theta_{Y}=\theta_{B}=0.02$. Each dataset consisted of L=500, 2,000, and 8,000 loci, with S=16 sequences per species per locus, and with the sequence length to be 500 sites. The number of replicate datasets was 10.

The data were then analyzed using bpp under the double-BDI model (fig. 6) to estimate the 14 parameters. We use gamma priors $\tau_{0}∼G(2,400)$ for the root age with the mean to be the true value (0.005), and θ∼G(2,200) with the mean 0.01 (true values are 0.005 and 0.02). We used a burn-in of 32,000 iterations, and then took $5\times 10^{5}$ samples, sampling every two iterations. Analysis of each dataset took ∼10 h for L=500 and ∼130 h for L=8,000, using eight threads on a server. The MCMC samples were processed to remove label-switching problems before they were summarized to approximate the posterior distribution.

### Simulation under a BDI Model with Poorly Separated Towers

We simulated a small dataset, with L=500 loci, under the BDI model of figure 1*a*, with $\left(\varphi_{X},\varphi_{Y})=(0.7,0.2\right)$ (see supplementary table S1, Supplementary Material online for the true values of all parameters). As $\varphi_{X}$ and $\varphi_{Y}$ were not far away from $\frac{1}{2}$ and the dataset was small, the posterior of the parameters was expected to be diffuse, and the posterior modes for parameters involved in the label-switching (or the two unidentifiable towers) to be poorly separated, posing a challenge to our relabeling algorithms.

We assigned gamma priors $\tau_{0}∼G(2,200)$ for the root age with the mean to be the true value (0.01), and θ∼G(2,400) with the mean 0.005 (true values are 0.002 and 0.01). We used a burn-in of 32,000 iterations, and then took $2\times 10^{5}$ samples, sampling every 10 iterations. We ran the same analysis twice to confirm consistency between runs, after the MCMC samples were processed to remove label switching.

## Supplementary Material

msac083_Supplementary_Data

## Data Availability

The relabelling algorithms are implemented in a C program, available at https://github.com/abacus-gene/bpp-msci-D-process-mcmc/. They are also implemented in BPP version 4.5 or later, available at https://github.com/bpp/bpp. The data files for the *Heliconius* datasets and the BPP control files for simulating and analyzing data under the BDI and double-BDI models (fig. 8) are available as an archive at http://abacus.gene.ucl.ac.uk/ziheng/data.html.
