# Supplementary figures and images for: Estimation of Cross-Species Introgression Rates Using Genomic Data Despite Model Unidentifiability
Source: Mol Biol Evol. 2022 Apr 13;39(5):msac083. doi: 10.1093/molbev/msac083 (PMC9087891; doi:10.1093/molbev/msac083)

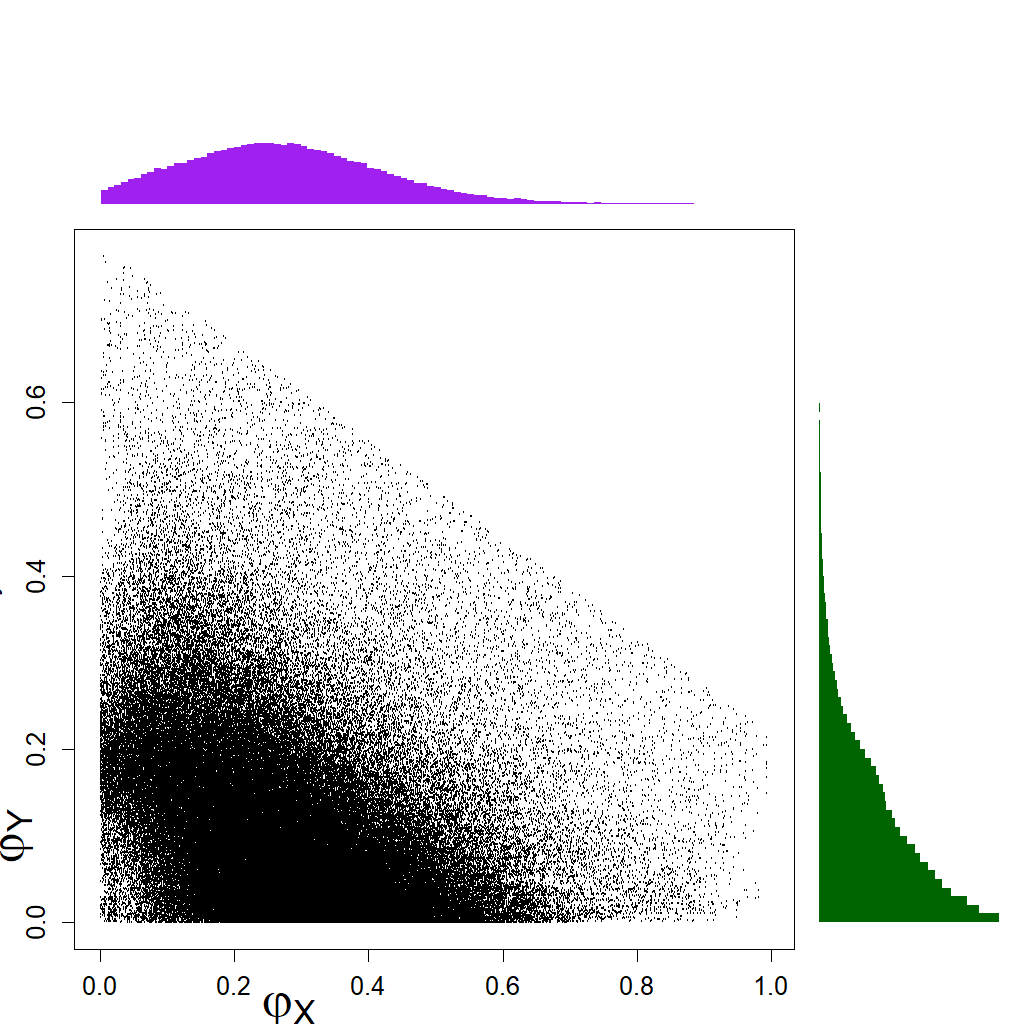

Supplement: msac083_Supplementary_Data [file msac083_supplementary_data.zip › fig-heliconius-exonic-L500-processed-CoG0-scatter.png]

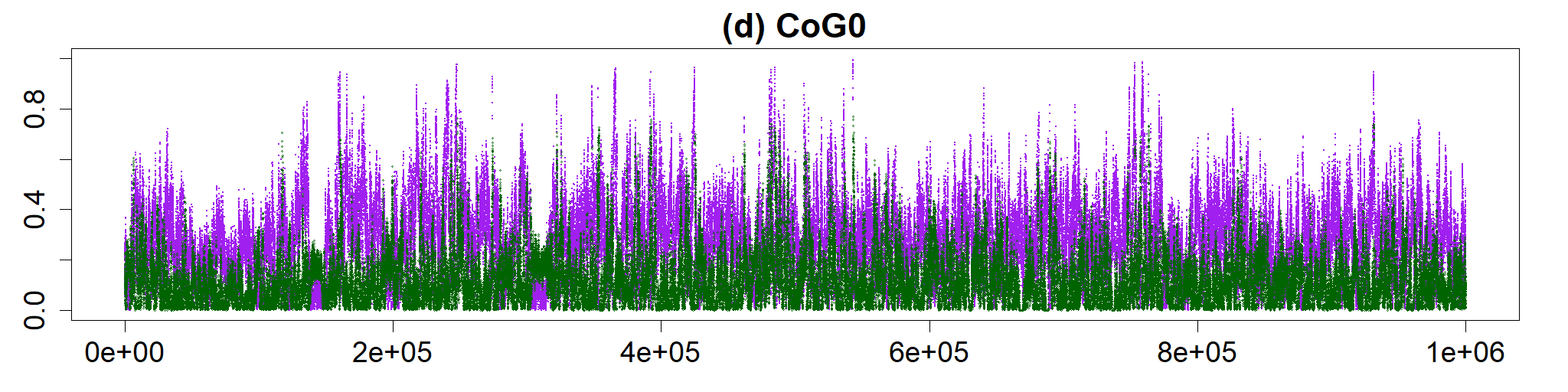

Supplement: msac083_Supplementary_Data [file msac083_supplementary_data.zip › fig-heliconius-exonic-L500-processed-CoG0-trace.png]

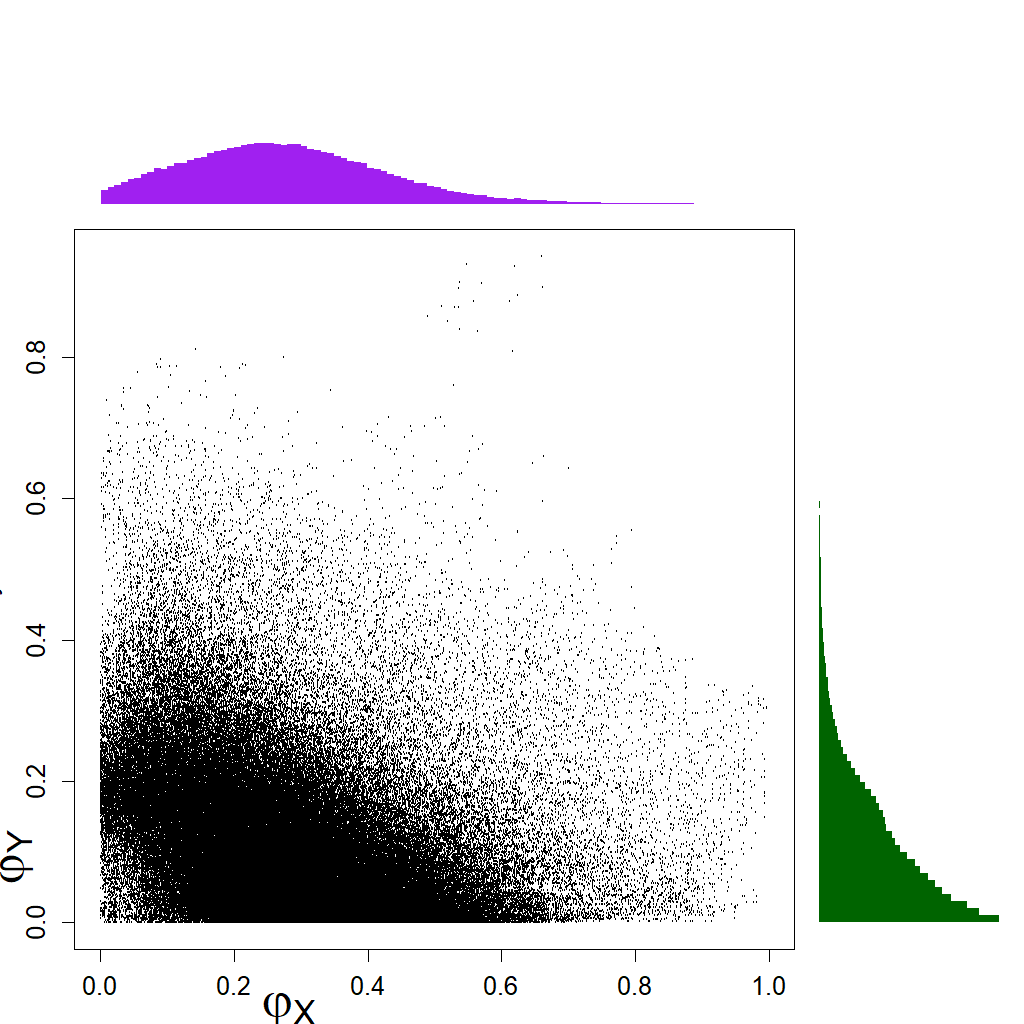

Supplement: msac083_Supplementary_Data [file msac083_supplementary_data.zip › fig-heliconius-exonic-L500-processed-CoGN-scatter.png]

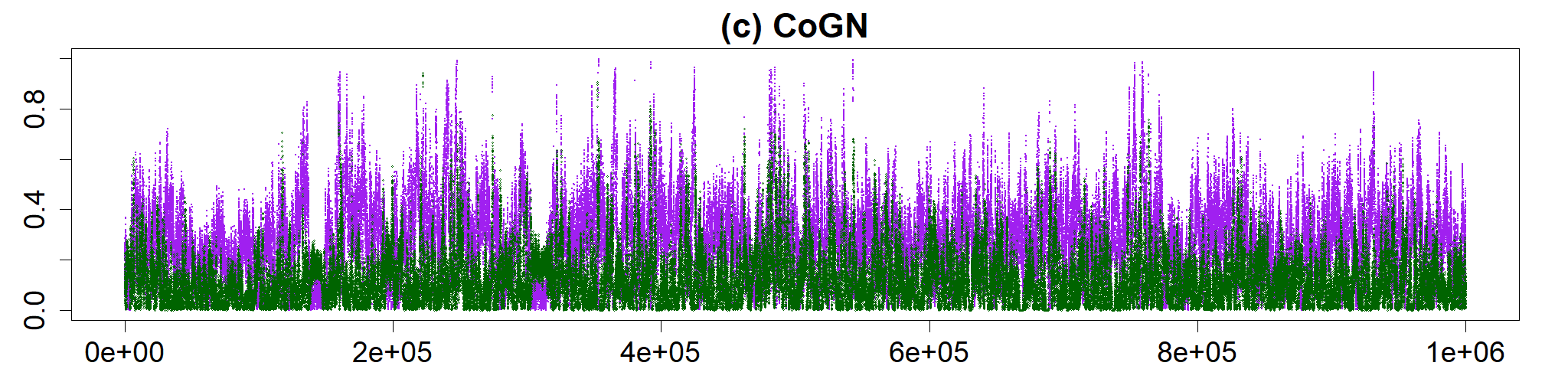

Supplement: msac083_Supplementary_Data [file msac083_supplementary_data.zip › fig-heliconius-exonic-L500-processed-CoGN-trace.png]

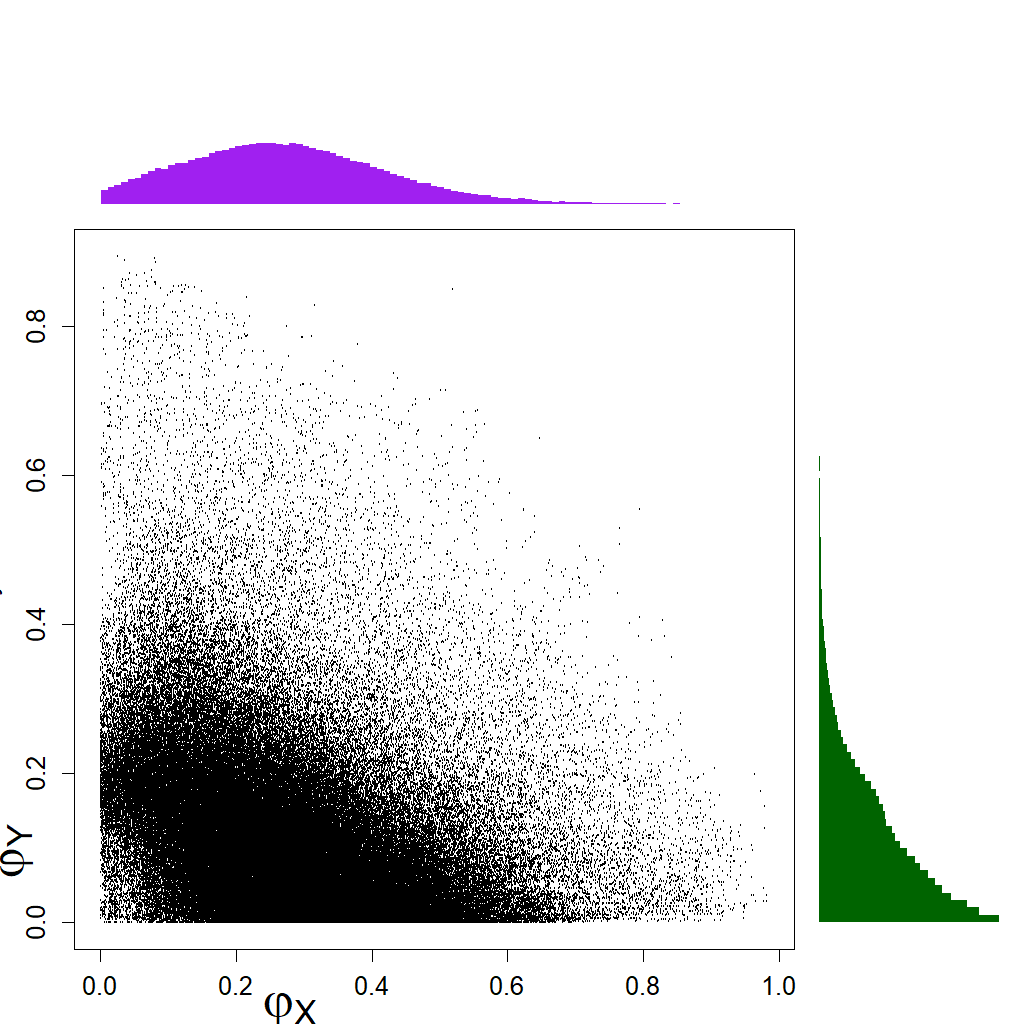

Supplement: msac083_Supplementary_Data [file msac083_supplementary_data.zip › fig-heliconius-exonic-L500-processed-scatter.png]

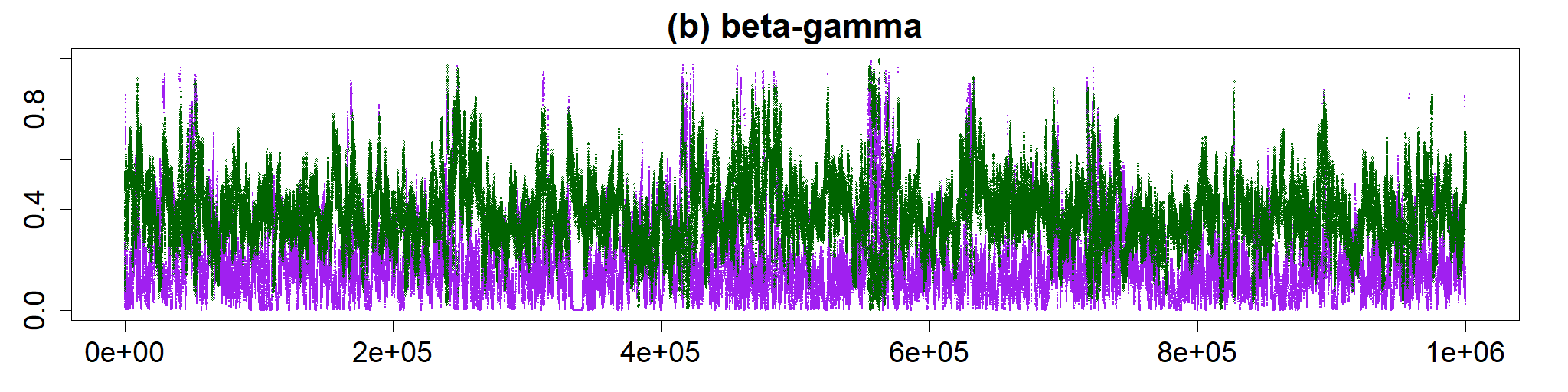

Supplement: msac083_Supplementary_Data [file msac083_supplementary_data.zip › fig-simulation-DD-L500-processed-trace.png]

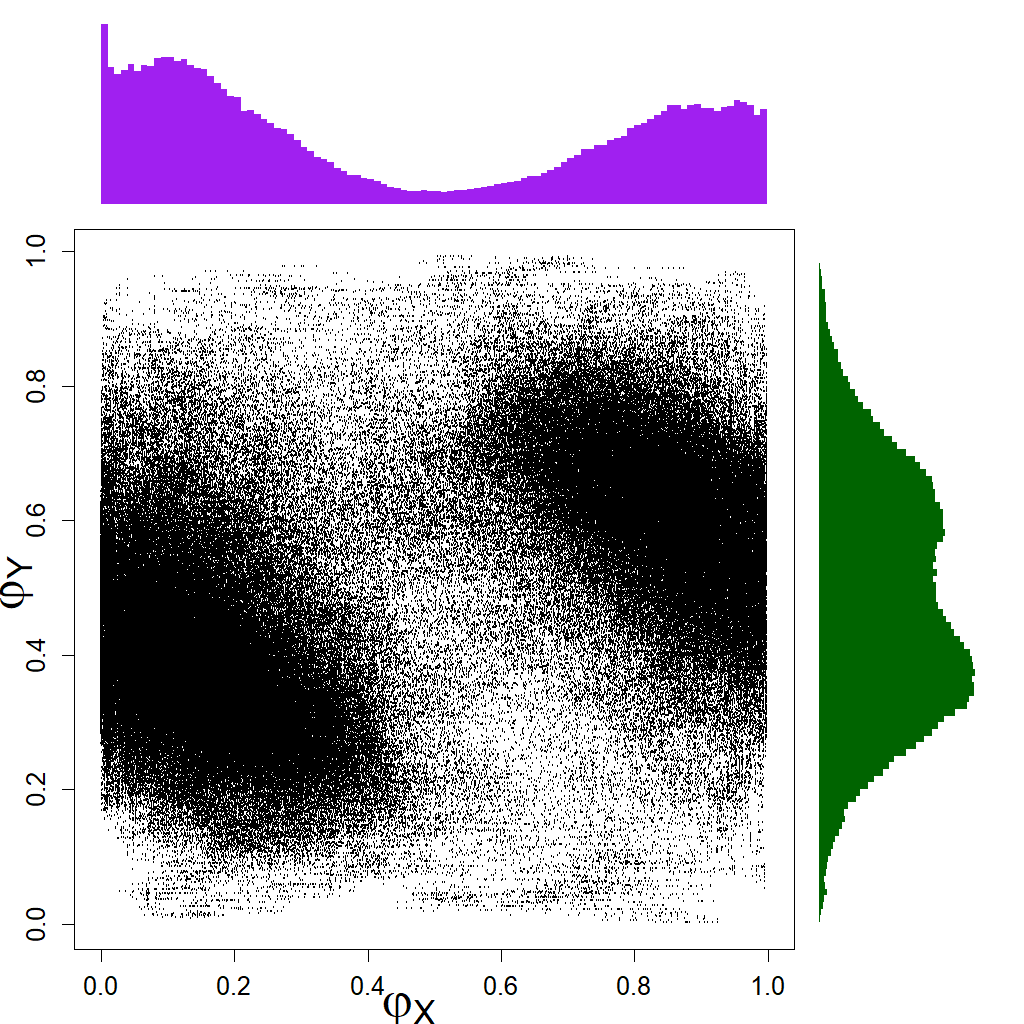

Supplement: msac083_Supplementary_Data [file msac083_supplementary_data.zip › fig-simulation-DD-L500-scatter.png]

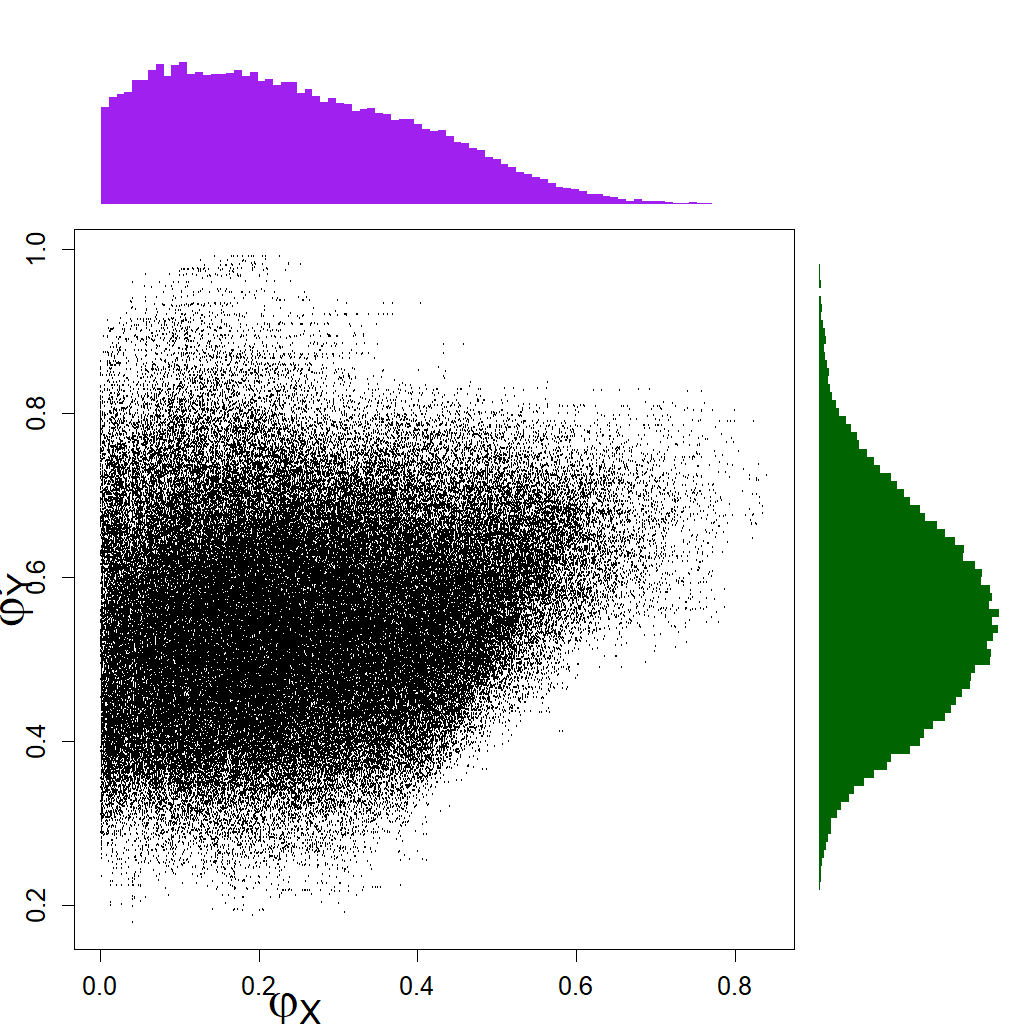

Supplement: msac083_Supplementary_Data [file msac083_supplementary_data.zip › fig-simulation-D-L500-processed-scatter.png]

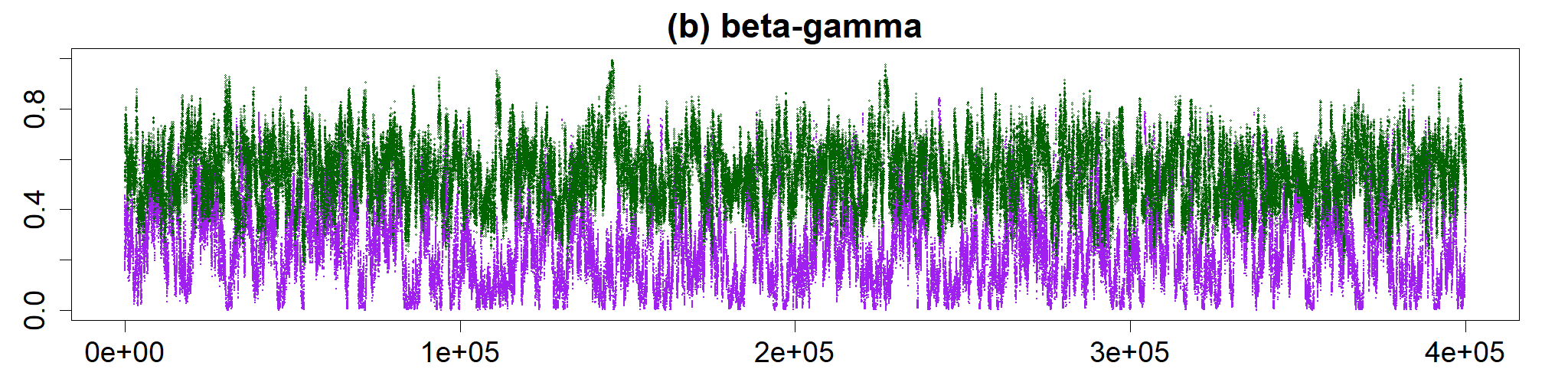

Supplement: msac083_Supplementary_Data [file msac083_supplementary_data.zip › fig-simulation-D-L500-processed-trace.png]

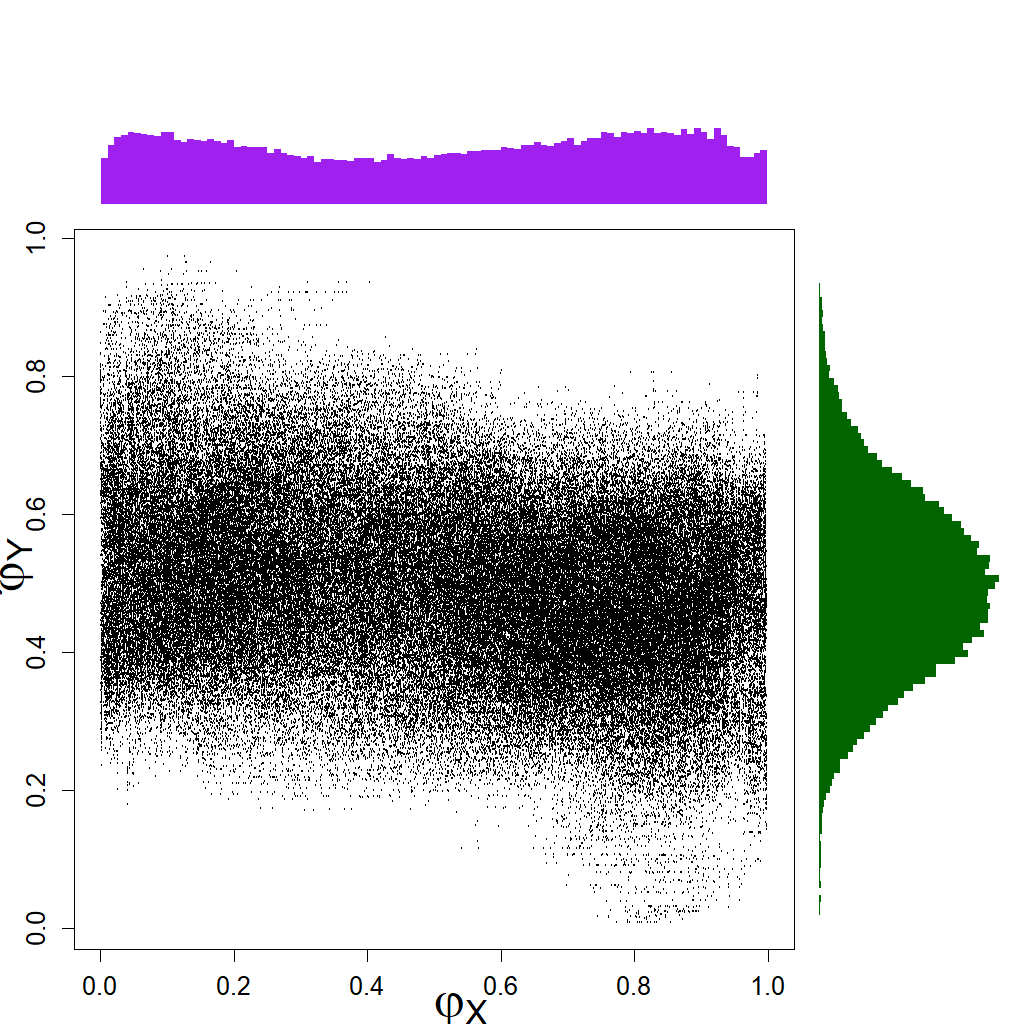

Supplement: msac083_Supplementary_Data [file msac083_supplementary_data.zip › fig-simulation-D-L500-scatter.png]

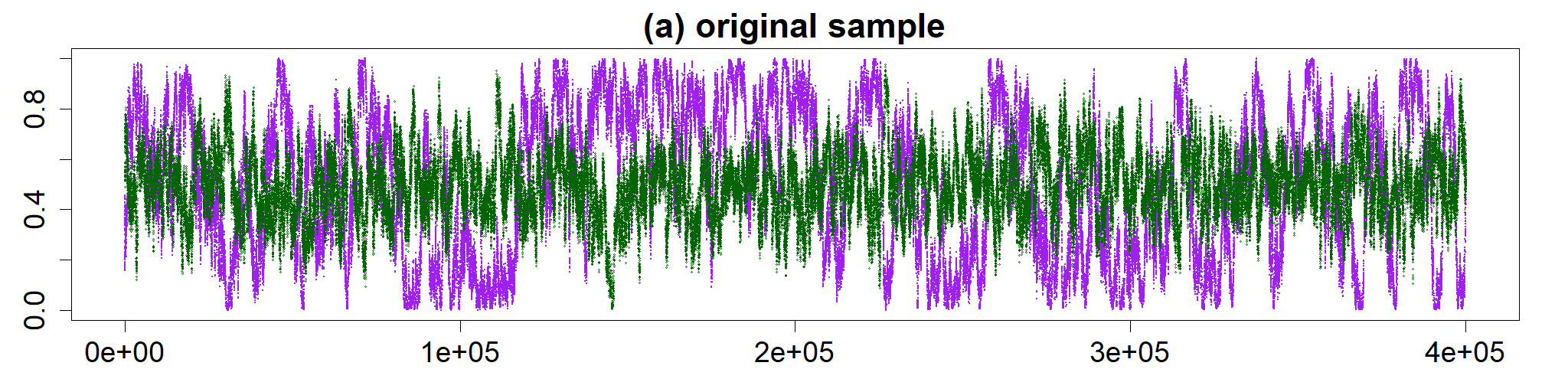

Supplement: msac083_Supplementary_Data [file msac083_supplementary_data.zip › fig-simulation-D-L500-trace.png]
